# Supplementary material for: Identification of clinical and ecological determinants of strain engraftment after fecal microbiota transplantation using metagenomics
Source: Cell Rep Med. 2022 Aug 4;3(8):100711. doi: 10.1016/j.xcrm.2022.100711 (PMC9418803; doi:10.1016/j.xcrm.2022.100711)
Supplement: Document S1. Figures S1–S5 and Text S1 [file mmc1.pdf]

Cell Reports Medicine, Volume *3*

Supplemental information

Identification of clinical and ecological determinants of strain engraftment after fecal microbiota transplantation using metagenomics

Daniel Podlesny, Marija Durdevic, Sudarshan Paramsothy, Nadeem O. Kaakoush, Christoph Högenauer, Gregor Gorkiewicz, Jens Walter, and W. Florian Fricke

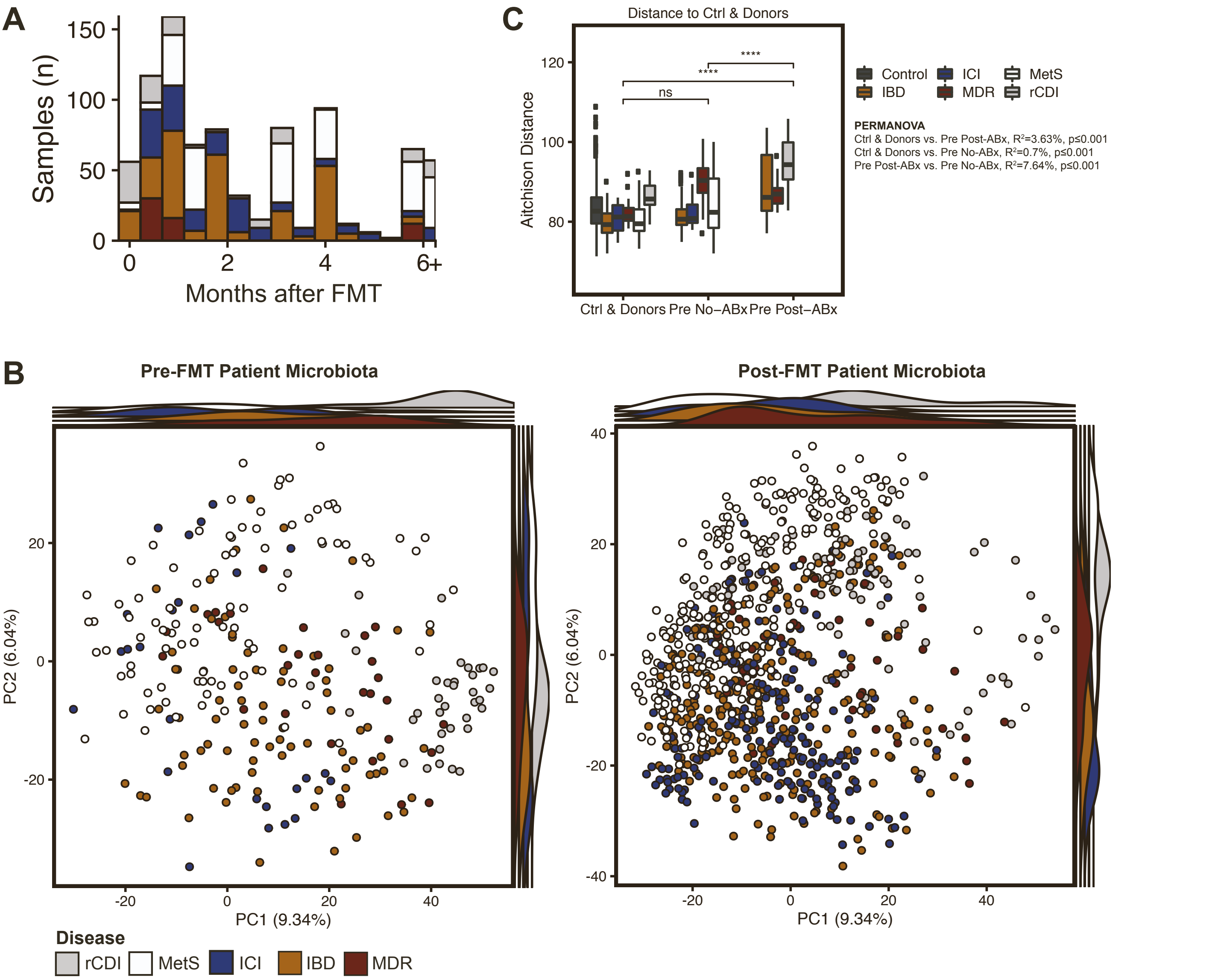

Figure S1. Overview of FMT samples between disease categories. Related to Figure 1. A. Available post-FMT samples and the time points of their collection. Histograms show the number of post-FMT samples that were collected during different time periods after FMT, colored by disease categories. B. Patient taxonomic microbiota composition across disease conditions. Taxonomic microbiota compositions of Pre-FMT and Post-FMT patients based on principal component analysis (PCA) of centered log-ratio-transformed relative species abundance, colored by disease background. Pre-FMT time points include only samples collected from patients before antibiotic treatments as part of the patient preparation for FMT, although rCDI patients received antibiotics due to their disease history. C. Taxonomic microbiota composition before and after antibiotic pretreatment. P-values based on the pairwise Wilcoxon test of Aitchison distances to Control and Donor samples (left) with FDR (BH) correction for multiple hypothesis testing, and PERMANOVA between groups (perm=999, adonis function, right). Asterisks denote significance thresholds: *p≤.01, **p≤.001, ***p≤.0001.

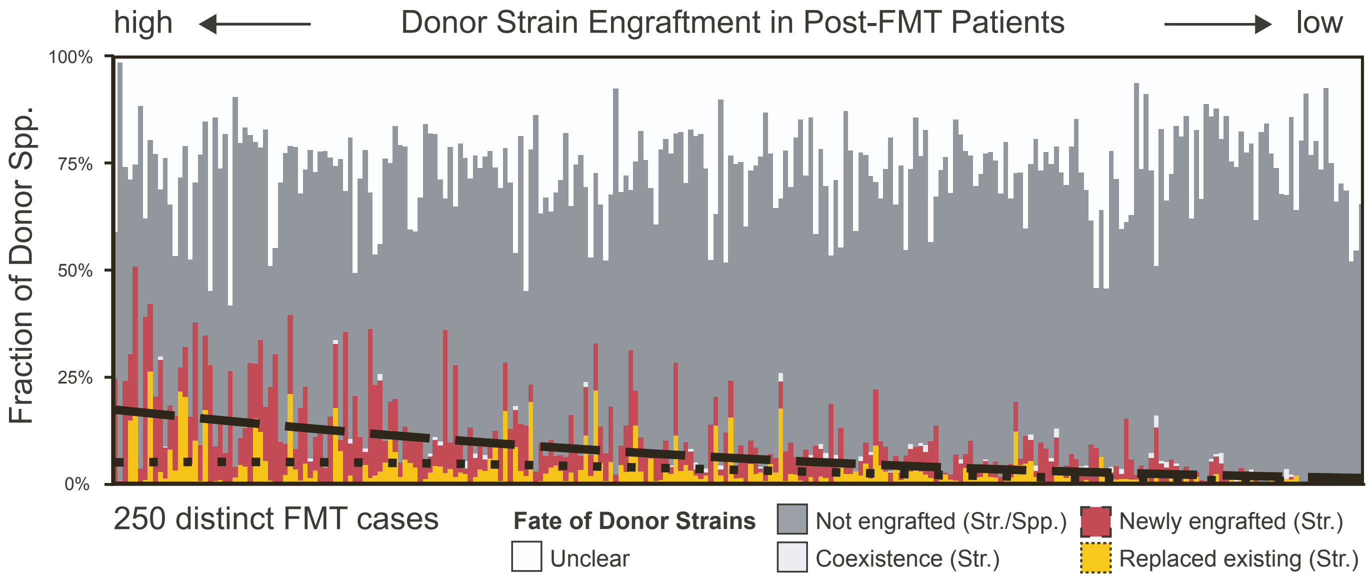

Figure S2. Fate of donor strains after FMT treatment. Related to Figure 3. Fate of donor strains after FMT treatment as the fraction of species detected in the donor microbiota, showing the same 250 FMT cases and sorting as depicted in Fig. 3A. Strains from the majority of donor species did not engraft in the patient (dark gray), and engrafted donor strains more often represented newly introduced species (red, dashed line) than replaced patient strains from a species that pre-existed in the patient before FMT (yellow, dotted line). Lines show binomial smoothing of the Fraction of Donor Spp. (glm, logit link).

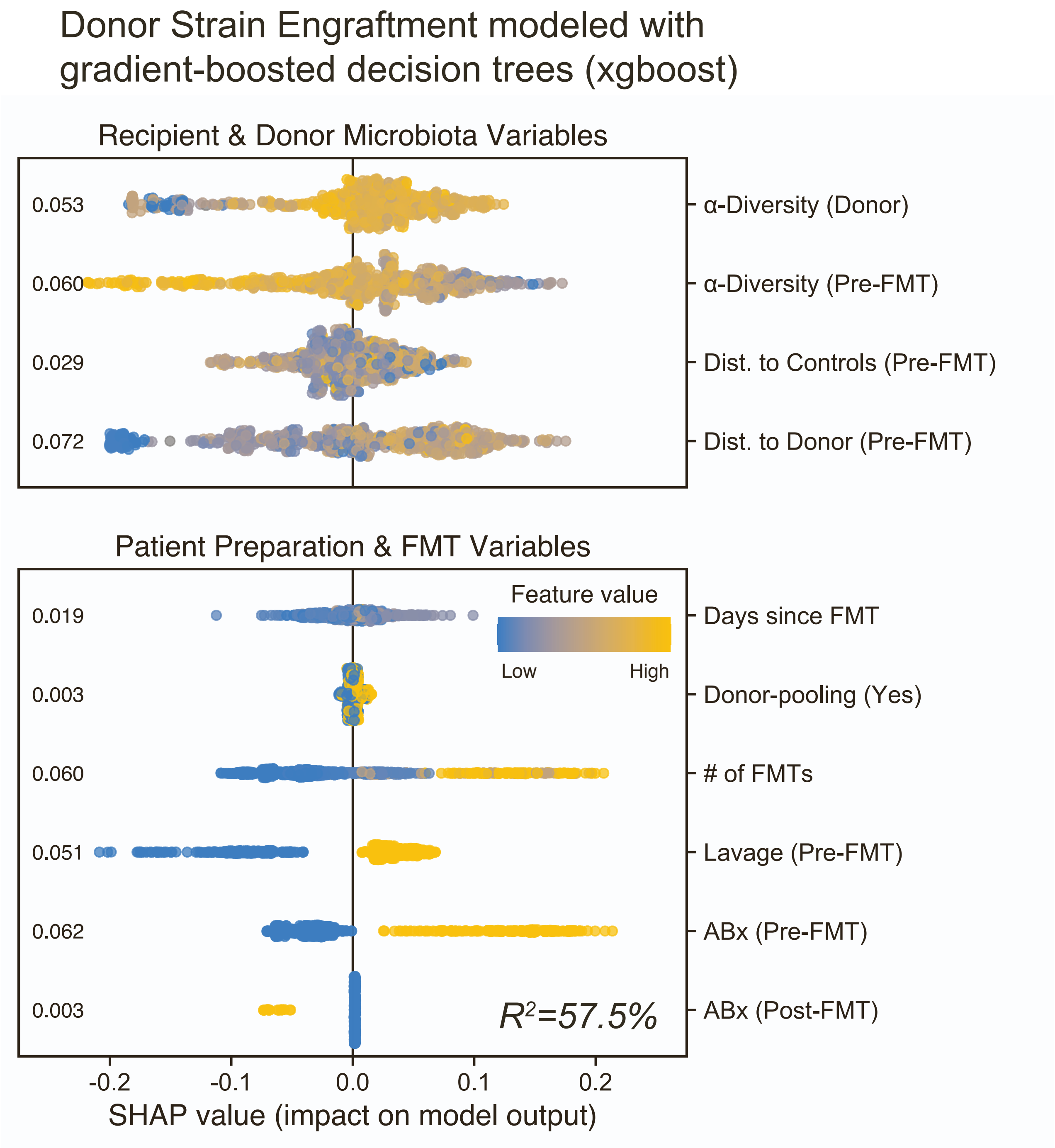

Figure S3. Gradient-boosted decision trees predict donor strain engraftment. Related to Figure 4. Gradient-boosted decision trees implemented in xgboost were applied to model donor strain engraftment as shown in Fig. 4. Data split into training and test set (80/20% split) was further blocked for individual cases to avoid information leakage during training. Model predictions based on fivefold cross-validation correlated strongly with hold-out data (r=0.76, p<.001). SHAP (Shapley additive explanations) values were calculated to visualize feature impact on model output.

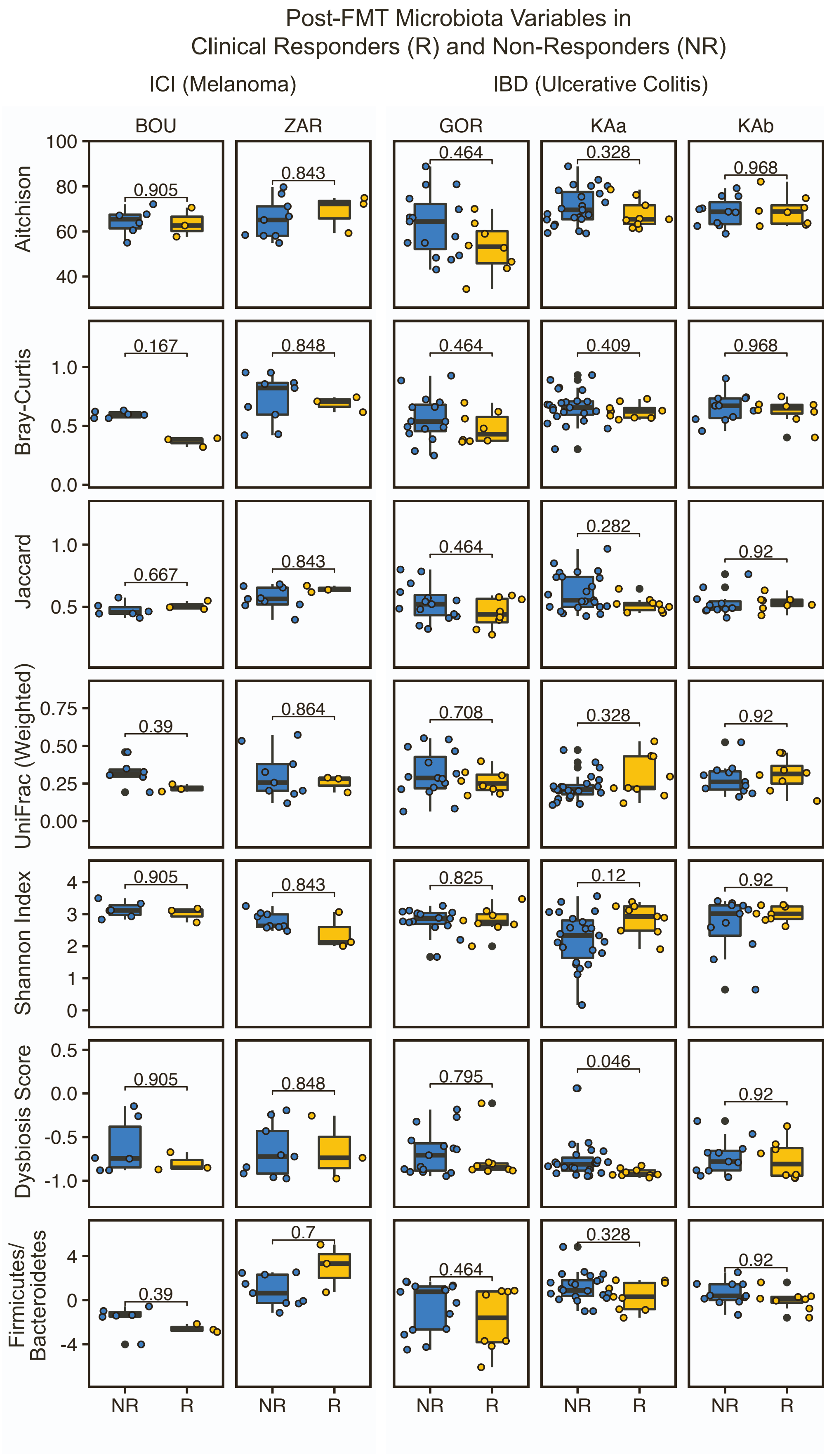

Figure S4. Microbiota comparisons between responders (R) and non-responders (NR) in ICI and IBD trials. Related to Figure 6. α-diversity (Shannon Index), Dysbiosis Score, the Firmicutes/Bacteroidetes ratio, and β-diversity (Aitchison distance, Bray-Curtis dissimilarity, Jaccard distance, weighted UniFrac) between patients and their donors for Responder/Non-Responder patient groups (see Methods) within the ICI and IBD studies. P-values based on the Wilcoxon test with FDR (BH) correction for multiple hypothesis testing within studies.

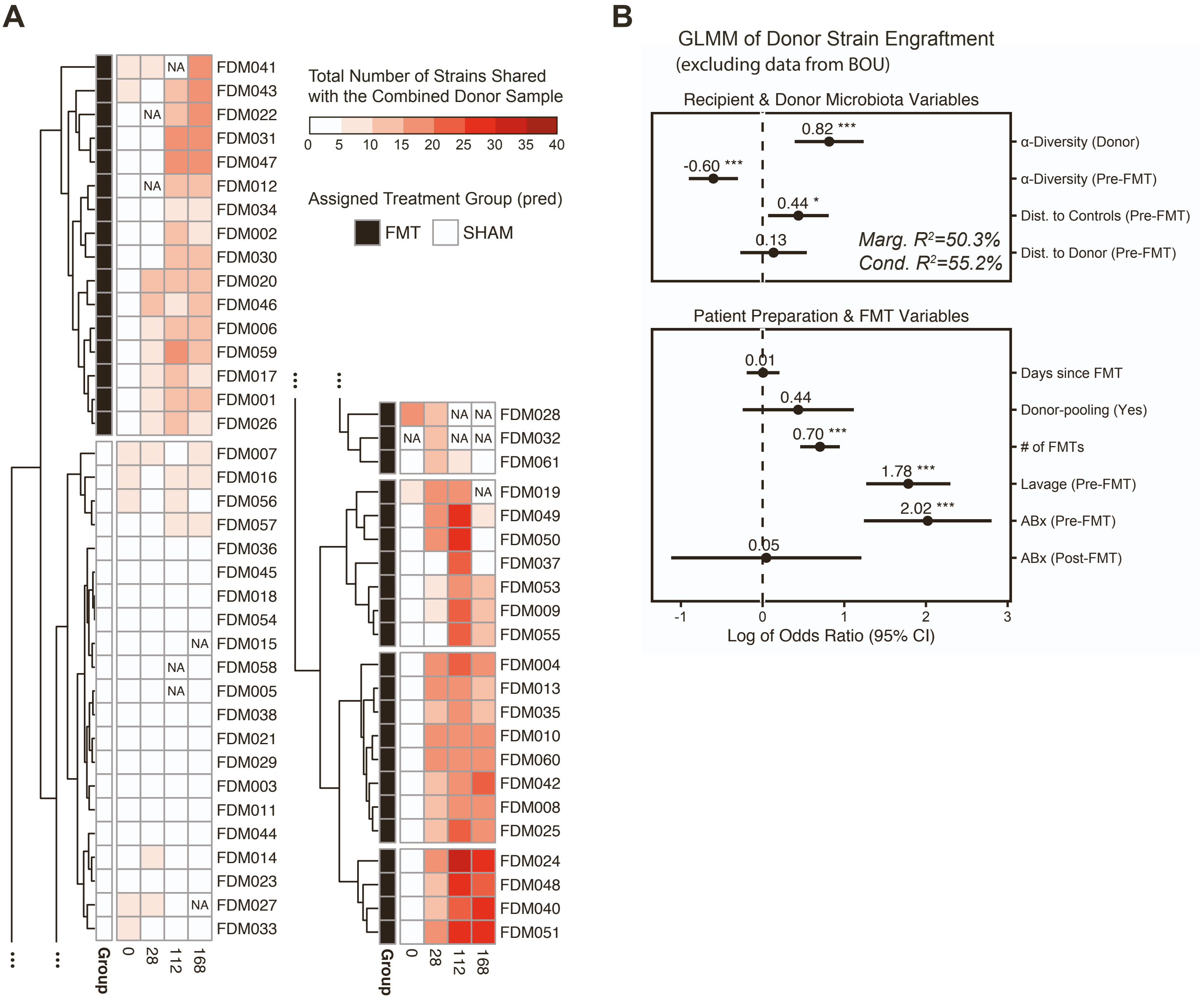

Figure S5. Data validation for the inclusion of FMT studies with missing information. Related to STAR Methods "Identification of recipient and donor-derived strains in post-FMT patients". A. Clustering of all samples from Ng et al. [1] based on shared strain profiles in order to determine the assignment of patients to the FMT or sham treatment groups. Hierarchical clustering of strain-sharing profiles between a concatenated donor sample (combined from five published donor metagenomes) and each patient from Ng et al. at one pre-FMT (0) and three post-FMT (28, 112, 168) time points highlights 21 patients whose samples share very few (mostly <5) strains with any donor sample. Since treatment assignments were not disclosed by Ng et al., these 21 patients were excluded from the analysis as they most likely belong to the sham treatment group of the study. **B.** GLMM of donor strain engraftment excluding data from Baruch et al. [2] Baruch et al. (BOU) collected pre-FMT samples before pretreatment with antibiotics. As for this dataset patient α-diversity and β-diversity to controls and donors was unavailable for the pre-FMTABx- sample, which would be most relevant for our model, the mean of those values that were observed in all other antibiotically treated pre-FMT patients was used instead to generate the model shown in Fig. 4. Here we generated a similar model without the data from Baruch et al., showing very similar results (odds ratio and significance) as in Fig. 4.

Supplementary Text S1. Influence of antibiotic used for patient pretreatment and route and number of FMT applications on donor strain engraftment. Related to Figure 3.

Different antibiotic pretreatments had distinct effects on post-FMT donor strain engraftment: MDRABx+ patients treated with antibiotics specific for Gram-negative bacteria (colistin and neomycin) acquired smaller proportions of donor-derived strains (HUT, 40.1 ± 33.6% of 209 strains) than IBDABx+ patients treated with an antibiotic cocktail against Gram-positive bacteria, parasites and fungi (vancomycin, paromomycin, and nystatin) (GOR, 94.4 ± 8.2% of 601 strains) or ICIABx+ patients treated with vancomycin and neomycin (BOU, 85.6 ± 15.7% of 232 strains) (Fig. 3C). The antibiotic treatment against Gram-positive bacteria in the IBDABx+ patients, which was associated with increased donor strain engraftment after FMT, also presented with enhanced dysbiosis before FMT (see GOR and HUT in Fig. 2).

Other forms of patient pretreatment, as well as the route of FMT application, also affected post-FMT microbiota assembly. Donor-derived strain contributions were larger in the KAa subgroup of FMT-treated IBD patients that received bowel preparation by lavage, followed by colonoscopic FMT and multiple enemas (75.6 ± 21.3% of 357 strains), compared to the KAb subgroup of the same study [3], which was treated with multiple enemas alone, without lavage and colonoscopic FMT (59.7 ± 27.5% of 298 strains) (Fig. 3C).

SUPPLEMENTAL REFERENCES

1. Ng, S.C., Xu, Z., Mak, J.W.Y., Yang, K., Liu, Q., Zuo, T., Tang, W., Lau, L., Lui, R.N., Wong, S.H., et al. (2021). Microbiota engraftment after faecal microbiota transplantation in obese subjects with type 2 diabetes: a 24-week, double-blind, randomised controlled trial. Gut.
2. Baruch, E.N., Youngster, I., Ben-Betzalel, G., Ortenberg, R., Lahat, A., Katz, L., Adler, K., Dick- Necula, D., Raskin, S., Bloch, N., et al. (2021). Fecal microbiota transplant promotes response in immunotherapy-refractory melanoma patients. Science 371, 602–609.
3. Paramsothy, S., Nielsen, S., Kamm, M.A., Deshpande, N.P., Faith, J.J., Clemente, J.C., Paramsothy, R., Walsh, A.J., van den Bogaerde, J., Samuel, D., et al. (2019). Specific Bacteria and Metabolites Associated With Response to Fecal Microbiota Transplantation in Patients With Ulcerative Colitis. Gastroenterology 156, 1440–1454.e2.
