# Supplementary material for: Identification of clinical and ecological determinants of strain engraftment after fecal microbiota transplantation using metagenomics
Source: Cell Rep Med. 2022 Aug 4;3(8):100711. doi: 10.1016/j.xcrm.2022.100711 (PMC9418803; doi:10.1016/j.xcrm.2022.100711)
Supplement: Document S2. Article plus supplemental information [file mmc8.pdf]

Cell Reports Medicine

Article

Identification of clinical and ecological determinants of strain engraftment after fecal microbiota transplantation using metagenomics

Graphical abstract

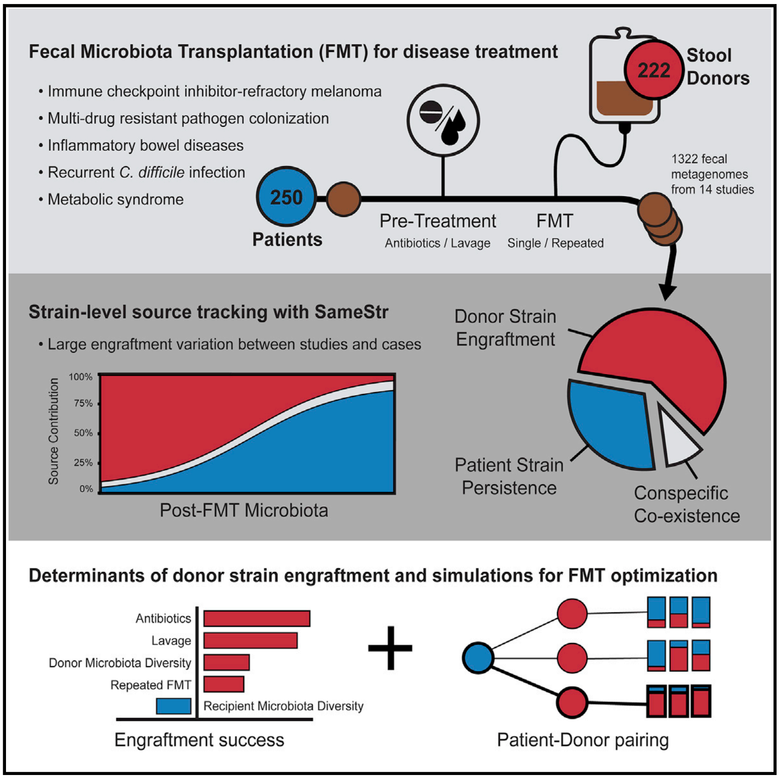

Highlights

- Pre-FMT patient preparation has larger impact on strain engraftment than disease
- Patient antibiotic pretreatment and lavage increase post-FMT donor strain engraftment
- Models indicate leverage for personalized FMT optimization with patient-donor pairing
- Engraftment rates not different in responders/non-responders with IBD or melanoma

Authors

Daniel Podlesny, Marija Durdevic, Sudarshan Paramsothy, ..., Gregor Gorkiewicz, Jens Walter, W. Florian Fricke

Correspondence

daniel.podlesny@uni-hohenheim.de (D.P.), w.florian.fricke@uni-hohenheim.de (W.F.F.)

In brief

Using strain-level microbiota profiling in patients treated with fecal microbiota transplantation (FMT), Podlesny et al. identify a mechanistic framework for post-FMT microbiome assembly that aligns with ecological theory. Donor strain engraftment is dependent on microbiome and clinical parameters, including antibiotic treatment and bowel lavage, independently of the treated disease.

Podlesny et al., 2022, Cell Reports Medicine *3*, 100711
August 16, 2022 © 2022 The Author(s).
https://doi.org/10.1016/j.xcrm.2022.100711

CellPress

Cell Reports Medicine

CellPress
OPEN ACCESS

Article

Identification of clinical and ecological determinants of strain engraftment after fecal microbiota transplantation using metagenomics

Daniel Podlesny,[1,*] Marija Durdevic,[2,3] Sudarshan Paramsothy,[4,5] Nadeem O. Kaakoush,[6] Christoph Högenauer,[2,3,7] Gregor Gorkiewicz,[2,3,8] Jens Walter,[9] and W. Florian Fricke[1,10,11,*]

[1]Department of Microbiome Research and Applied Bioinformatics, University of Hohenheim, Stuttgart, Germany
[2]Institute of Pathology, Medical University of Graz, Graz, Austria
[3]Theodor Escherich Laboratory for Medical Microbiome Research, Medical University of Graz, Graz, Austria
[4]Department of Gastroenterology and Hepatology, Concord Repatriation General Hospital, Sydney, NSW, Australia
[5]Concord Clinical School, University of Sydney, Sydney, NSW, Australia
[6]School of Medical Sciences, UNSW Sydney, Sydney, NSW, Australia
[7]Division of Gastroenterology and Hepatology, Department of Internal Medicine, Medical University of Graz, Graz, Austria
[8]BioTechMed, Interuniversity Cooperation, Graz, Austria
[9]APC Microbiome Ireland, School of Microbiology and Department of Medicine, University College Cork, Cork, Ireland
[10]Institute for Genome Sciences, University of Maryland School of Medicine, Baltimore, MD, USA
[11]Lead contact
*Correspondence: daniel.podlesny@uni-hohenheim.de (D.P.), w.florian.fricke@uni-hohenheim.de (W.F.F.)
https://doi.org/10.1016/j.xcrm.2022.100711

SUMMARY

Fecal microbiota transplantation (FMT) is a promising therapeutic approach for microbiota-associated pathologies, but our understanding of the post-FMT microbiome assembly process and its ecological and clinical determinants is incomplete. Here we perform a comprehensive fecal metagenome analysis of 14 FMT trials, involving five pathologies and >250 individuals, and determine the origins of strains in patients after FMT. Independently of the underlying clinical condition, conspecific coexistence of donor and recipient strains after FMT is uncommon and donor strain engraftment is strongly positively correlated with pre-FMT recipient microbiota dysbiosis. Donor strain engraftment was enhanced through antibiotic pretreatment and bowel lavage and dependent on donor and recipient α-diversity; strains from relatively abundant species were more likely and from predicted oral, oxygen-tolerant, and gram-positive species less likely to engraft. We introduce a general mechanistic framework for post-FMT microbiome assembly in alignment with ecological theory, which can guide development of optimized, more targeted, and personalized FMT therapies.

INTRODUCTION

Fecal microbiota transplantation (FMT) infuses the microbiota of healthy donor feces into the intestinal tract of a patient in the clinical attempt to treat a microbiota-associated disease.[1] Although there are several mechanisms by which FMT might work,[2] much focus has been devoted to resolve disturbances of the gut microbial ecosystem, which are often referred to as “dysbiosis.”[3] FMT has been validated in randomized controlled trials as an efficient treatment for recurrent *Clostridioides difficile* infection (rCDI), with a success rate of ~90% that surpasses those of conventional antibiotic treatments.[4,5] For the inflammatory bowel disease (IBD) ulcerative colitis, FMT has been less effective in inducing remission (24%–32% versus 5%–9% for placebo), but clinical response rates surpassed those reported in phase III clinical trials for several biological agents (golimumab and vedolizumab).[2] Modest benefits have also been reported for the treatment of insulin sensitivity in subjects suffering from obesity and metabolic syndrome with FMT.[6] In addition, two recent trials suggest a potential role for FMT in cancer therapy, demonstrating re-induction of a response to anti-PD-1 immunotherapy in ~30% of immune checkpoint inhibitor (ICI)-refractory melanoma patients (n = 25) after FMT from patients who had previously responded to ICI.[7,8] FMT is currently being tested for numerous other microbiota-associated infectious, inflammatory, and metabolic diseases. Yet, the mechanism of action of FMTs and their specific short- and long-term effects on the recipient microbiota remain poorly understood, even for established clinical indications.[9,10] Furthermore, FMT represents an intriguing experimental approach to establish causality for the association of the human microbiota with specific pathologies, which are currently mostly studied using fecal transplantation into rodents, which are limited in their ability to replicate the human setting.[11]

The dynamics of post-FMT microbiota organization and the clinical, host, and ecological factors that govern microbiome assembly in different FMT-treated populations and at the level of individual patients and microbes have not been extensively

This is an open access article under the CC BY license (http://creativecommons.org/licenses/by/4.0/).

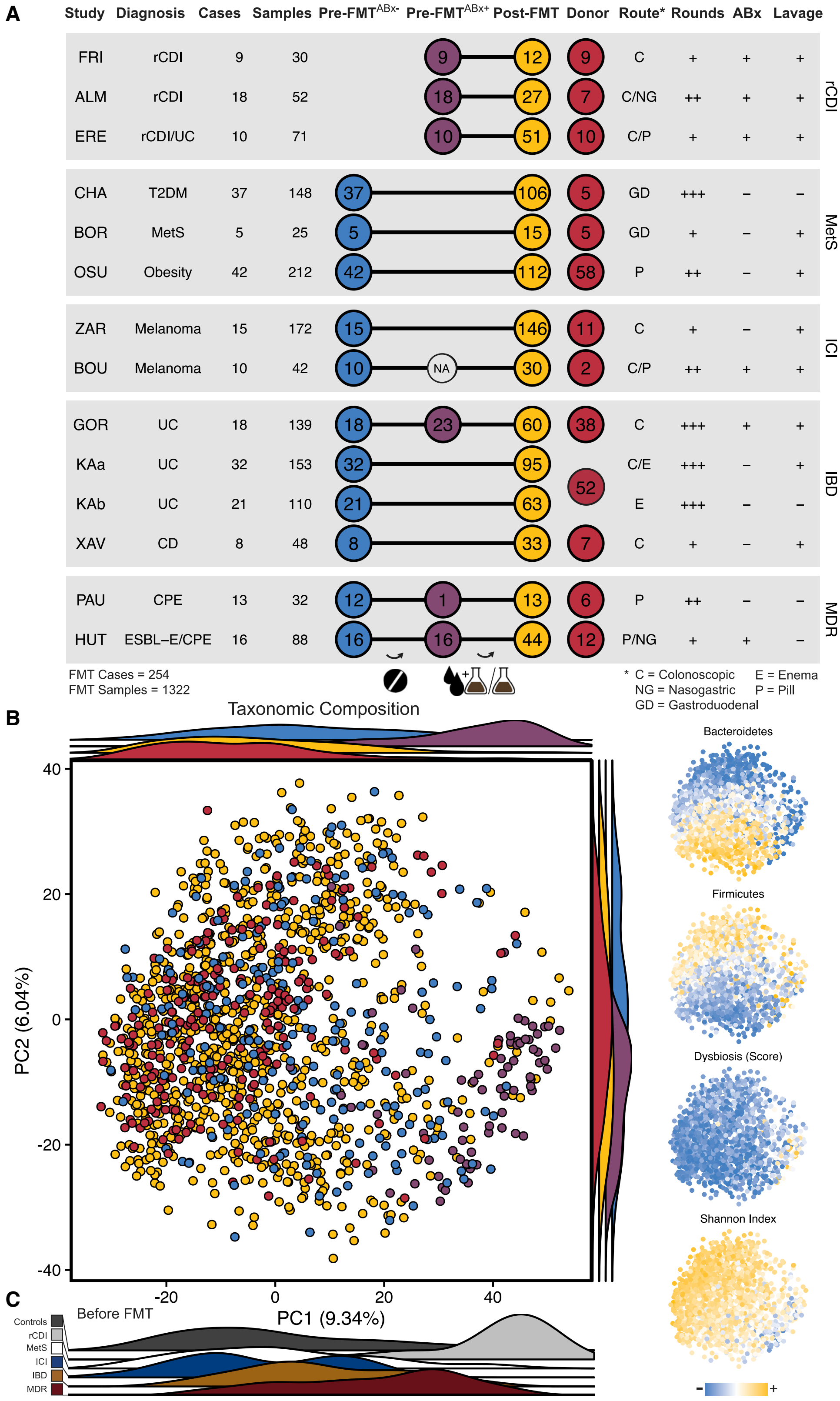

(legend on next page)

studied. Previous studies mostly relied on 16S rRNA analysis, which lacks the taxonomic resolution to track specific recipient and donor-derived microbiota members after FMT. This limits our ability to elucidate the ecological principles that govern post-FMT microbiota assembly, as interactions between microbiome members are hypothesized to be most competitive among closely related taxa, including conspecific strains belonging to the same species.[12] However, previous strain-level microbiota analyses came to differing conclusions about conspecific strain populations after FMT, reporting "durable coexistence of donor and recipient strains" after FMT in metabolic syndrome patients,[13] or engraftment of multi-strain donor species populations in an "all-or-nothing manner" without substantial and lasting coexistence of recipient and donor strains.[14] Furthermore, frequent coexistence of donor and recipient strains has been justified with the importance of neutral and stochastic ecological determinants,[15] whereas another study identified competition and adaptation that would favor fitness as a main ecological driver for microbiome organization after FMT.[16] These ambiguous conclusions reflect our limited understanding of the ecological principles that govern the post-FMT microbiome assembly process and its relationship to the clinical conditions in which FMT is applied.

Although in many aspects FMT represents an ecosystem restoration approach, ecological framework theories have only recently been applied to understand and predict FMT outcomes.[17] Although the healthy adult gut microbiota exhibits colonization resistance to invading microbes that compete for ecological niches that are shared with resident microbes,[18,19] a dysbiotic microbiota (e.g., after antibiotic treatment) provides reduced colonization resistance to *C. difficile* infection[20] and is unable to recover in rCDI patients due to repeated rounds of unsuccessful antibiotic treatments.[21] In rCDI patients, this reduced colonization resistance may be responsible for the establishment of a transplanted donor microbiota after FMT.[14,22] Yet, to what extent a transplanted donor microbiota can colonize patients with other pathologies that do not present with severely disrupted gut microbiomes remains unclear.

In this study, we characterize post-FMT microbiome assemblies at the strain level in patients who present with a range of disease and treatment backgrounds. We develop generalizable models to predict and control FMT outcomes, determine the relationship between post-FMT microbiota assembly and clinical response, thereby defining the opportunities and limitations of FMT-based precision microbiota therapies and outlining clinical strategies to optimize them.

RESULTS

Fecal microbiota changes and dysbiosis signatures in a human meta-cohort of FMT-treated patients with different medical conditions

To compare the effect of FMT on fecal microbiota composition in patients with different medical conditions, a comprehensive meta-cohort was assembled of metagenomic shotgun sequence data from FMT-treated patients and donors, including 1,322 samples from 254 complete cases (Table S1). This FMT meta-cohort (Figure 1A) includes patients treated for recurrent *C. difficile* infection (rCDI, three studies), the IBDs ulcerative colitis (2 studies) and Crohn disease (one study); metabolic syndrome, type 2 diabetes mellitus, or obesity (MetS, three studies); drug-resistant *Enterobacteriaceae* carriage (MDR, two studies); or immune checkpoint inhibitor-refractory melanoma (ICI; two studies). Post-FMT samples were relatively evenly distributed between patient groups over the first 4 months after FMT (Figure S1A). For comparison, a control cohort of healthy individuals from several unrelated fecal microbiome studies was included (see STAR Methods). There were substantial differences between FMT study protocols, including the use of bowel lavage and antibiotic treatment ($ABx^{+/-}$), the FMT application type (by nasoduodenal or colonic endoscopy, enema, or capsule), as well as the number of FMTs received (which varied between a single and up to 41 applications). All rCDI patients were treated with antibiotics as part of their therapeutic regimens, while each of the ICI, IBD, and MDR groups included at least one study in which antibiotics were used as a bowel cleansing strategy to deplete the resident microbiota and prepare patients for donor microbiota engraftment (Figure 1A). This meta-cohort reflects the current state and heterogeneity of patient populations and disease backgrounds that have been experimentally treated with FMT and provides an opportunity to identify the shared mechanistic and ecological foundation of post-FMT microbiome assembly.

We applied principal-component analysis of a β-diversity distance computed with centered log ratio-transformed relative species abundances to characterize the variation in fecal microbiota composition over the entire cohort (Figure 1B). The separation of samples along PC1 correlated with α-diversity (Shannon diversity, $r = -0.48$, $p < 0.0001$) and a dysbiosis score ($r = 0.30$, $p < 0.0001$) based on relative species abundances,[23] whereas PC2 primarily reflected shifts in the *Bacteroidetes*/*Firmicutes* ratio between samples (Figure 1B, inlet, $r = -0.72$, $p < 0.0001$). Before but not after FMT, rCDI patient samples clustered separately from other patient samples (Figures 1C, S1B and S1C); the pre-FMT microbiota of patients with other diseases, who were not pretreated with

Figure 1. Overview and taxonomic microbiota composition of the FMT meta-cohort

(A) Overview of treatment modalities, number and distribution of cases, and samples for 13 studies (Table S1) from five conditions included in the FMT meta-cohort.

(B) Taxonomic microbiota compositions based on principal-component analysis (PCA) of centered log-ratio-transformed relative species abundance. Samples in the main plot are categorized and color-coded as pre-FMT^{ABx-} (blue), antibiotically pretreated pre-FMT^{ABx+} (purple), and post-FMT (yellow) patient and donor (red) samples. Ridgeline density plots show sample distributions along the two first principal components based on the same categories. In the small plots (right side) samples from the same PCA are color-coded based on (from top to bottom) scaled cumulative relative abundances of *Bacteroidetes*, *Firmicutes*, dysbiosis score,[23] and α-diversity (Shannon index).

(C) Ridgeline density plots showing only pre-FMT (untreated and antibiotically pretreated) patient samples, colored by disease category. The MDR studies included patients with a history of antibiotic treatments, even before the beginning of the FMT trials.

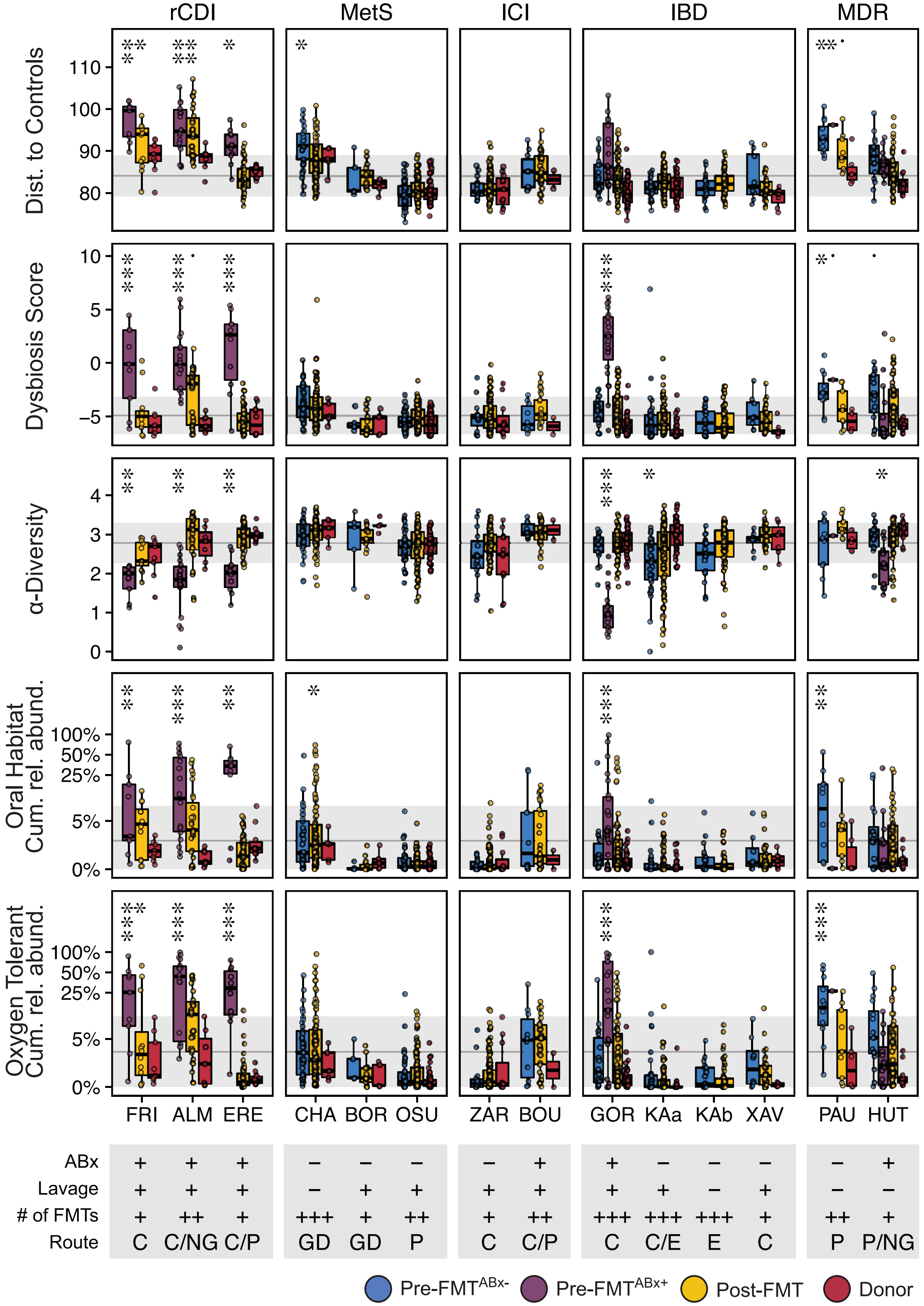

(legend on next page)

antibiotics showed a similar taxonomic composition as the microbiota of donors and healthy controls (Figures 1B and 1C). This finding shows that only rCDI but none of the other medical conditions before antibiotic treatment were associated with a pronounced dysbiosis. Antibiotic treatment placed IBD and MDR patient samples before FMT in the dysbiotic range of rCDI patient samples (Figure 1B). Similarly, β-diversity analysis (Aitchison distance) shows a significant difference in taxonomic microbiota compositions between pre-FMT patients who received antibiotics, but not treatment-naive pre-FMT patients and healthy controls (Figure S1C, PERMANOVA, $p \leq 0.001$). Given that rCDI patents receive repeated antibiotic treatment attempts to eradicate *C. difficile* infection,[24] our analysis suggests that antibiotics are the main drivers of the compositional microbiota variations shown on PC1 and result in similar alterations to the microbiota, independently of the patient's medical condition.

To further characterize dysbiosis in the FMT meta-cohort, pretreated and naive patient and donor samples were separately compared based on taxonomic and functional microbiota parameters. $rCDI^{ABx+}$ patient samples consistently exhibited significantly decreased α-diversity, increased taxonomic distance relative to healthy controls, and elevated dysbiosis scores compared with ICI^{ABx-}, IBD^{ABx-}, and, with the exception of one study, $MetS^{ABx-}$ cohort patients (Figure 2 generalized linear mixed model, $p \leq 0.001$). However, IBD^{ABx+} patients from one study (GOR[25]) exhibited a dysbiosis state comparable to rCDI patients after broad-spectrum (vancomycin, paromomycin, and nystatin) antibiotic pretreatment (Figure 2). A similar trend was observed in MDR patients, although these patients were difficult to classify, as they included patients with previous antibiotic treatments, following 48 h of antibiotic discontinuation (PAU[26]) or received antibiotics specific for only gram-negative bacteria (HUT, colistin and neomycin;[27]). Dysbiosis was also characterized by increased cumulative relative abundances of oral and oxygen-tolerant species in $rCDI^{ABx+}$, IBD^{ABx+}, and MDR^{ABx+} patient samples (Figure 2, see STAR Methods for details on species classification). Dysbiosis signatures were resolved or at least improved in rCDI patients and absent from all other patient populations after FMT. Thus, microbiota-associated diseases besides rCDI that have been experimentally treated with FMT are characterized by low levels of dysbiosis, but dysbiosis is induced after antibiotic treatment.

Strain tracking reveals variable, patient- and treatment-dependent, donor strain contributions to the post-FMT microbiota

The pretreatment microbiome of FMT recipients was dominated by species that were shared with their donors and that, on average, accounted for >75% cumulative relative abundance (Figure 3A, inlet). In order to determine the origins of specific members of the post-FMT patient microbiota, as well as the relative contributions of donor and recipient-derived, as well as newly detected strains, a subspecies taxonomic microbiota profiling was carried out with the SameStr tool.[22,28] SameStr defines strains as unique subspecies lineages that are shared between related microbiome samples with the potential for strain persistence or transfer, as opposed to more widely shared subspecies lineages that could also be present in unrelated microbiomes.[22] This analysis allowed us to unambiguously assign post-FMT patient strains to recipient (shared strains between pre- and post-FMT patient metagenomes), donor (shared strains between post-FMT patient and donor metagenomes), or other sources (newly detected strains, neither shared with pre-FMT patients nor donor metagenomes). Here, we analyzed strain-resolved transmission of microbes using data from 250 FMT cases (FRI = 9, ALM = 16, ERE = 10, CHA = 37, BOR = 5, OSU = 39, ZAR = 15, BOU = 10, GOR = 23, KAa = 32, KAb = 21, XAV = 8, PAU = 9, HUT = 16), excluding samples from patients who had been exposed to post-FMT antibiotic treatment (OSU = 9, ZAR = 5, PAU = 4).

To validate the specificity of the shared strain calls identified by SameStr for the FMT meta-cohort, we determined the "false-positive" shared strain detection rate in 2,606 sample pairs from distinct individuals who would not be expected to share or have exchanged microbial strains (both donors and post-FMT patients) (Figure 3B). This analysis identified only 0.4 ± 1.1 shared strains in unrelated sample pairs, attesting to the high specificity of SameStr for the identification of unique or FMT case-specific shared strains, which is needed to infer donor strain engraftment in post-FMT patients.

Most strains detected in patients after FMT could be assigned to either the donor or the recipient, or they represented new, i.e., previously undetected, strains or species from environmental or other sources (Figure 3A). The coexistence of donor and recipient-derived strains from the same species was detectable in 26.8% of post-FMT patients (n = 250 cases, using the latest available time point). However, these conspecific strains represented only 0.6% of all detected species in post-FMT patients (n = 21,404) and accounted for only 2.2% ± 5.5% cumulative relative abundance per patient. Moreover, recipient and donor strain coexistence was rare for all disease backgrounds and individual studies (Figure 3C). In FMT cases with the potential to produce conspecific strain competition after FMT, i.e., if the same bacterial species was detected in recipients and donors before FMT, most often a new, previously undetected strain

Figure 2. Taxonomic and functional microbiota comparison of FMT recipients, with or without antibiotic treatment, post-FMT patients, and donors in the different studies

Generalized linear mixed-effects models (GLMM, see STAR Methods) highlight differences in taxonomic and functional microbiota metrics between $pre\text{-}FMT^{ABx-}$ (blue), pretreated $pre\text{-}FMT^{ABx+}$ (purple), Post-FMT (yellow), and donor (red) samples relative to a reference cohort of 739 healthy adults (gray line and area denote mean ± SD, respectively), based on the average distance to healthy control samples (β-diversity, Aitchison distance), the dysbiosis score,[23] α-Diversity (Shannon index), and the cumulative relative abundance of oxygen-tolerant or oral bacterial species. Metadata abbreviations indicate pretreatment with ABx and lavage (+/−); single (+), two (++), or multiple (+++) FMTs; colonoscopic (C), nasogastric (NG), or gastroduodenal (GD) FMT route, enema (E), and pill/capsule (P) administration. Sample sizes (see STAR Methods for the reference cohort) are as shown in Figure 1A, excluding post-FMT samples from patients who have been exposed to antibiotics after FMT (OSU = 9, ZAR = 5, PAU = 4). Significant differences of the different sample types from healthy controls were determined for each metric and study separately with GLMMs. Asterisks denote significance thresholds: $p \leq 0.1$, $^{*}p \leq 0.01$, $^{**}p \leq 0.001$, $^{***}p \leq 0.0001$.

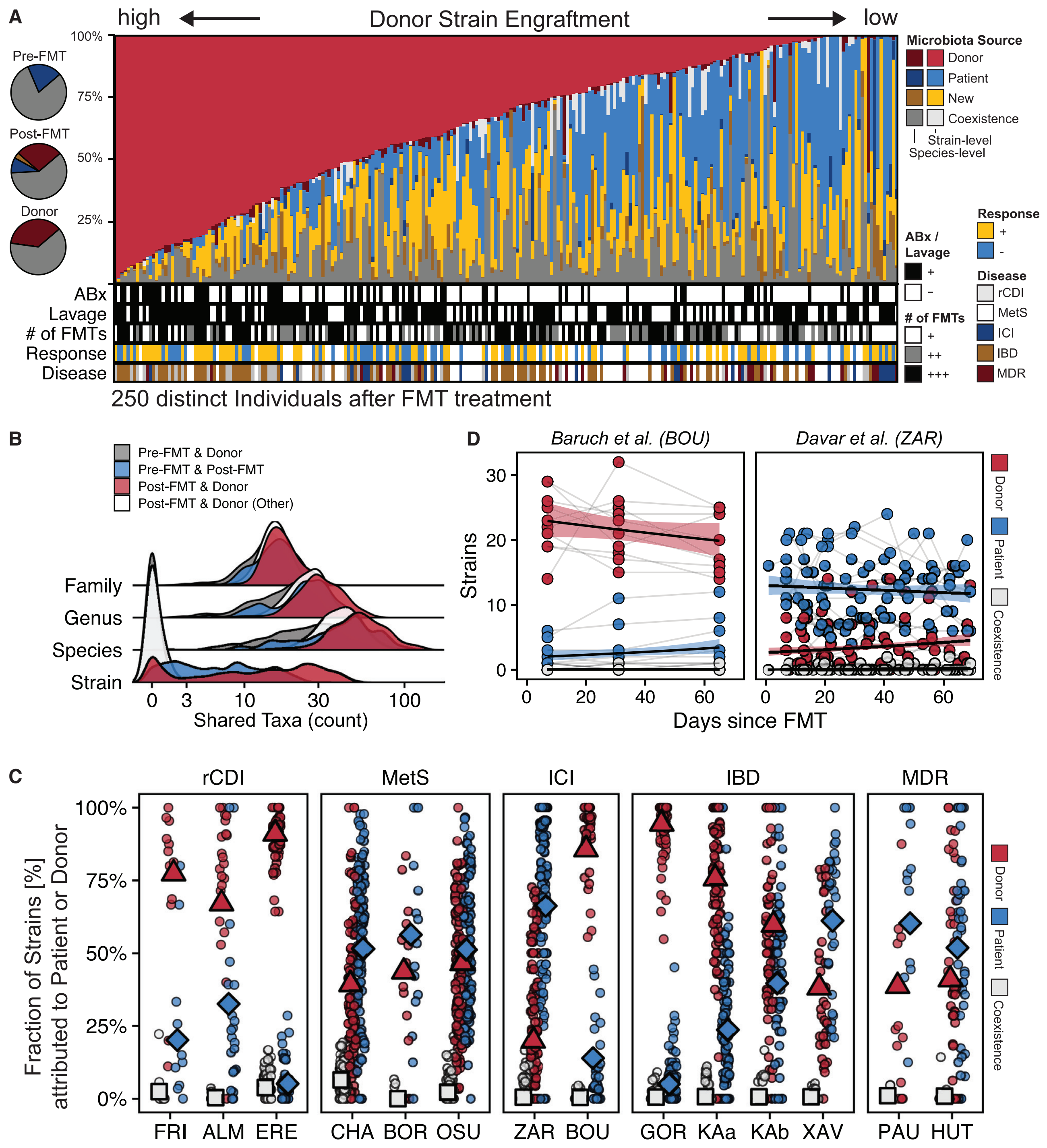

	FRI	ALM	ERE	CHA	BOR	OSU	ZAR	BOU	GOR	KAa	KAb	XAV	PAU	HUT
ABx	+	+	+	–	–	–	–	+	+	–	–	–	–	+
Lavage	+	+	+	–	+	+	+	+	+	+	–	+	–	–
# of FMTs	+	++	+	+++	+	++	+	++	+++	+++	+++	+	++	+
Route	C	C/NG	C/P	GD	GD	P	C	C/P	C	C/E	E	C	P	P/NG

(legend on next page)

(n = 2782, 36.2%), only the recipient strain (n = 1334, 17.3%), or only the donor strain (n = 965, 12.5%) were identified after FMT, but rarely coexistence of conspecific recipient and donor strains (n = 127, 1.7%). In the remaining cases, the species could no longer be detected (n = 1320, 15.2%), or the strain could not be resolved (n = 1166, 15.2%). The presence of distinct strains from the same species in recipients and donors before FMT therefore appears to predominantly lead to competitive exclusion during post-FMT microbiota assembly.

In the FMT meta-cohort, donor-derived strains accounted for the largest fraction of the post-FMT patient microbiota (18.8% ± 26.7%), followed by recipient-derived strains (12.2% ± 20.1%) (Figure 3A). However, donor- and recipient-derived strain and relative abundance fractions varied substantially between cases and studies (Figures 3A and 3C). While donors consistently contributed larger strain fractions to the rCDI patient microbiota after FMT than recipients (76.5% ± 27.1% of 787 detected donor and recipient-derived strains), the post-FMT microbiota of non-antibiotically pretreated MetS^{ABx-}, IBD^{ABx-}, and ICI^{ABx-} patients was dominated by recipient strains (Figure 3C). In contrast, donor-derived strains became dominant in post-FMT IBD^{ABx+} and ICI^{ABx+} patients from two trials that prepared patients with antibiotic treatments for FMT (Figure 3C). The antibiotic pretreatment effect on donor strain engraftment was particularly striking in ICI patients (Figure 3D), where donor-derived strains accounted for 85.6% ± 15.7% (n = 232 strains in total) in ICI^{ABx+} patients[8] compared with only 19.9% ± 23.5% (n = 180 strains in total) in ICI^{ABx-} patients.[7] In both ICI trials, donor- and recipient-derived, as well as coexisting, strain contributions to the post-FMT patient microbiota remained remarkably stable over at least 60 days after FMT (Figure 3D). Although the lack of available pre-FMT^{ABx+} microbiome data prevented a more comprehensive analysis, donor strain engraftment was affected by the antibiotic used for patient pretreatment, as well as the route and number of FMT applications (Figure 3C, see Text S1).

Donor strains from the majority of species (62.1% ± 15.4%) did not engraft in patients after FMT (Figure S4). Only 10.8% ± 9.6% of donor species were represented by a donor-derived strain in post-FMT patients and a larger fraction of these strains resulted in the introduction of a new species to the patient (7.3% ± 7.5% of all donor species) than the replacement of a pre-existing patient strain with a donor strain from the same species (3.2% ± 4.6% of all donor species), suggesting that donor strains are less likely to engraft by competing with a recipient strain from the same species than by filling an empty species niche in the recipient microbiota.

In summary, donor strain engraftment after FMT appears clinically expandable, including for different medical conditions, using antibiotic microbiota disruption to induce dysbiosis in preparation for FMT, as well as other FMT protocol parameters.

Microbiota and clinical determinants of donor strain engraftment after FMT in individual patients

In order to gain a conceptual understanding of the factors that determine donor microbiota engraftment after FMT in individual patients, we wanted to determine the effects of both ecological microbiota and clinical FMT parameters on post-FMT patient microbiota assembly. As ecological parameters, recipient and donor microbiota (α/β) diversity, and as clinical parameters, patient pretreatment (ABx, lavage) and FMT modalities (no. of FMTs) were used as input variables for a generalized linear mixed model (GLMM) to estimate donor-derived strain fractions after FMT per patient (Figure 4A, Tables S4 and S5). Gradient-boosted decision trees (xgboost) with 5-fold cross-validation were used to capture non-linear associations in the data and confirm the GLMM-based findings (Figure S3).

Of the microbiota variables, donor α-diversity (Shannon index) had the strongest estimated positive effect (odds ratio [OR] = 0.83, $p < 0.001$) on overall donor strain engraftment, whereas recipient α-diversity had an estimated negative effect (OR = −0.61, $p < 0.001$). Simulations with the GLMM to estimate the specific marginal effects of recipient and donor α-diversity, using alternative, artificial values for the Shannon index, i.e., the highest and lowest value that was detected in any donor from the cohort, indicated that a disproportionately large fraction of strains from high-diversity donors would engraft in low-diversity patients after FMT (Figure 4B). Compositional divergence (β-diversity, Aitchison distance) of the recipient relative to the control microbiota (healthy reference cohort), a microbiota marker for dysbiosis (Figure 2), was positively correlated with donor microbiota engraftment (OR = 0.46, $p < 0.05$). Compositional distance between recipient and donor microbiota, however, was not

Figure 3. Strain profiling in FMT recipients showed substantial variation in donor strain engraftment between studies that are linked to FMT treatment modalities

(A) Post-FMT microbiota relative abundance fractions contributed from donor (red), patient (blue), new (yellow), or coexisting (gray) strains, as detected in the last available post-FMT sample per patient. Darker colors refer to species-level relative abundance fractions if strains could not be resolved. *Left:* Circle chart showing average cumulative relative abundances of shared species in pre- and post-FMT patient and donor samples (gray) and between pre- and post-FMT patient (blue) and post-FMT patient and donor (red) samples.

(B) Validation of SameStr's specificity to infer donor strain engraftment from shared strain detection. Very few shared strains were identified between pre-FMT patient and donor (gray) and between unrelated post-FMT patient and donor (white) sample pairs, whereas strain sharing is frequent between pre- and post-FMT patient (blue) and between post-FMT patient and corresponding donor (red) sample pairs. The microbiota compositions of all sample pairs overlapped widely at higher taxonomic levels (family, genus, species).

(C) Comparison of donor-derived (red), recipient-derived (blue), and coexisting (gray) strain fractions in post-FMT patient samples (of the sum of donor and recipient-derived strains) between disease groups and individual studies from the meta-cohort. Symbols denote the mean value of the latest available post-FMT sample per patient and across all cases of a study. Study metadata are shown as follows: Antibiotic (ABx) and bowel lavage patient pretreatment: Yes (+), No (−); Number of FMTs: single (+), two (++), or multiple (+++) rounds; FMT application: by colon (C), nasogastric (NG), or gastroduodenal (GD) endoscopy, enema (E), or pill/capsule (P).

(D) Longitudinal comparison of donor-derived (red), patient-derived (blue), and coexisting (white) strain fractions in post-FMT samples from antibiotically pretreated (BOU) and non-pretreated (ZAR) ICI-refractory melanoma patients.

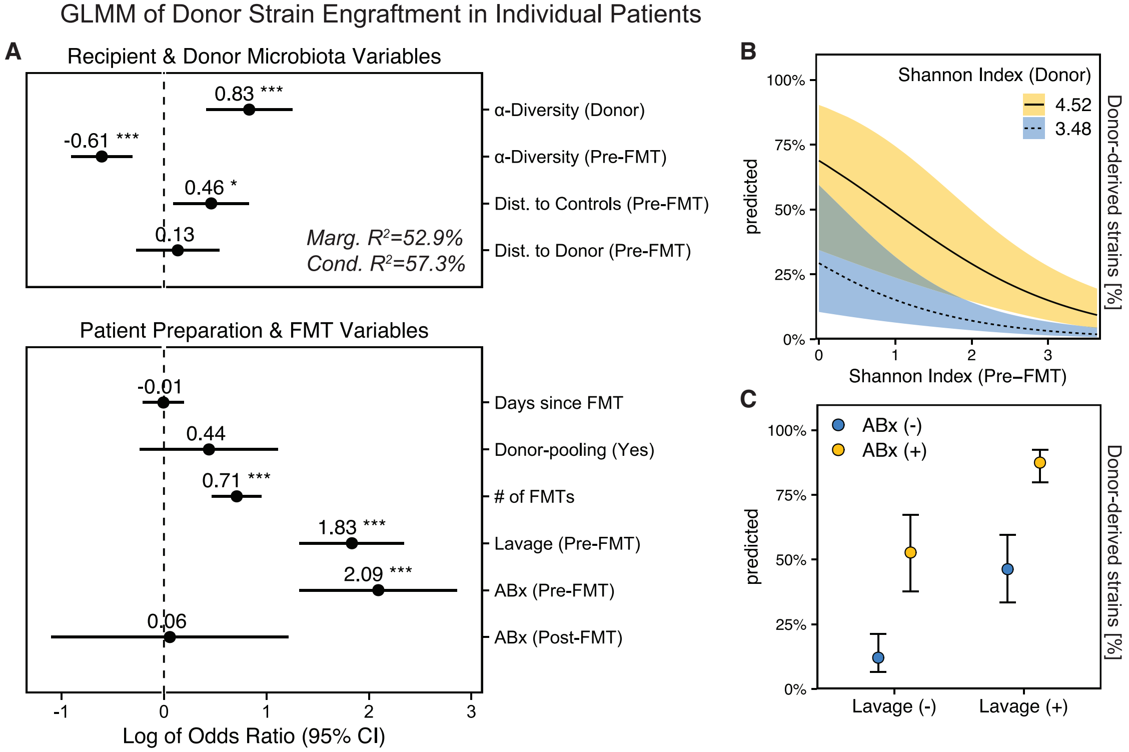

Figure 4. For individual patients, donor microbiota engraftment after FMT is dependent on patient and donor microbiota characteristics and clinical modalities of the FMT treatment

(A) Forest plot showing the relevance of microbiota and clinical parameters for donor-derived strain fractions in post-FMT patients in the FMT meta-cohort, as determined with a generalized linear mixed model. The model is based on data from 254 clinical FMT cases, including samples from post-FMT patients (samples n = 801; FRI = 12, ALM = 24, ERE = 51, CHA = 105, BOR = 15, OSU = 112, ZAR = 146, BOU = 30, GOR = 58, KAa = 95, KAb = 63, XAV = 33, PAU = 13, HUT = 44), their respective donors (n = 140), and earliest available pre-FMT samples (n = 254).

(B) Simulations with this model to determine the marginal effects of α-diversity on donor strain engraftment, i.e., using real values in combination with the minimum or maximum Shannon index detected in any donor in the FMT cohort (min/max within 95% confidence intervals), indicate a disproportionate impact of high-α-diversity donors on low-α-diversity FMT recipients.

(C) Similar simulations predict independent marginal effects of ABx and lavage pretreatment on donor strain engraftment. Shaded areas and bars denote the 95% confidence intervals (Wald). Asterisks denote significance thresholds: *p ≤ 0.01, **p ≤ 0.001, ***p ≤ 0.0001.

predicted to significantly affect engraftment (OR = 0.13, p = ns), suggesting that donor strain engraftment is more dependent on recipient dysbiosis and donor and recipient α-diversity than specific donor microbiota compositions.

Of the clinical variables, antibiotic pretreatment (OR = 2.09, p < 0.001), bowel lavage (OR = 1.83, p < 0.001), and multiple FMT applications (OR = 0.71, p < 0.001) were all predicted to increase donor strain engraftment based on our GLMM (Figure 4A). Time since FMT had no effect (OR = −0.01, p = 0.958), suggesting temporally stable donor contributions to the post-FMT microbiota. Donor sample pooling was also not predicted to increase donor strain engraftment (OR = 0.44, p = 0.202), which is surprising given the positive association of engraftment with donor α-diversity and could indicate that a mixture of multiple samples is not functionally equivalent for FMT to a single high-diversity sample. GLMM-based FMT outcome predictions using simulated antibiotic treatment and lavage while maintaining all other ecological and clinical parameters indicate that both pretreatment measures independently increase the rate of donor strain engraftment after FMT (Figure 4C).

In summary, post-FMT microbiota assembly appears to be driven both by the recipient (α/β-diversity) and donor (α-diversity) microbiota, as well as the FMT procedure, i.e., resident microbiota depletion before FMT (by ABx treatment and lavage) and repeated exposure of the patient to the donor microbiota (multiple FMTs).

Taxonomic determinants of individual donor strain engraftments

As the previous model estimated the community-wide (α/β-diversity) determinants of donor strain engraftment for individual patients, we next sought to compare the engraftment probabilities for individual strains from different bacterial taxa in a separate GLMM. This second model outlines the taxonomic and functional boundaries for donor strain engraftment, i.e., whether strains from specific taxa or with taxon-specific properties are more or less likely to engraft after FMT. For this GLMM, we used the same set of variables as described above, but in combination with relative abundance and other species features, such as Gram stain, oral habitat, spore formation, or oxygen

CellPress
OPEN ACCESS

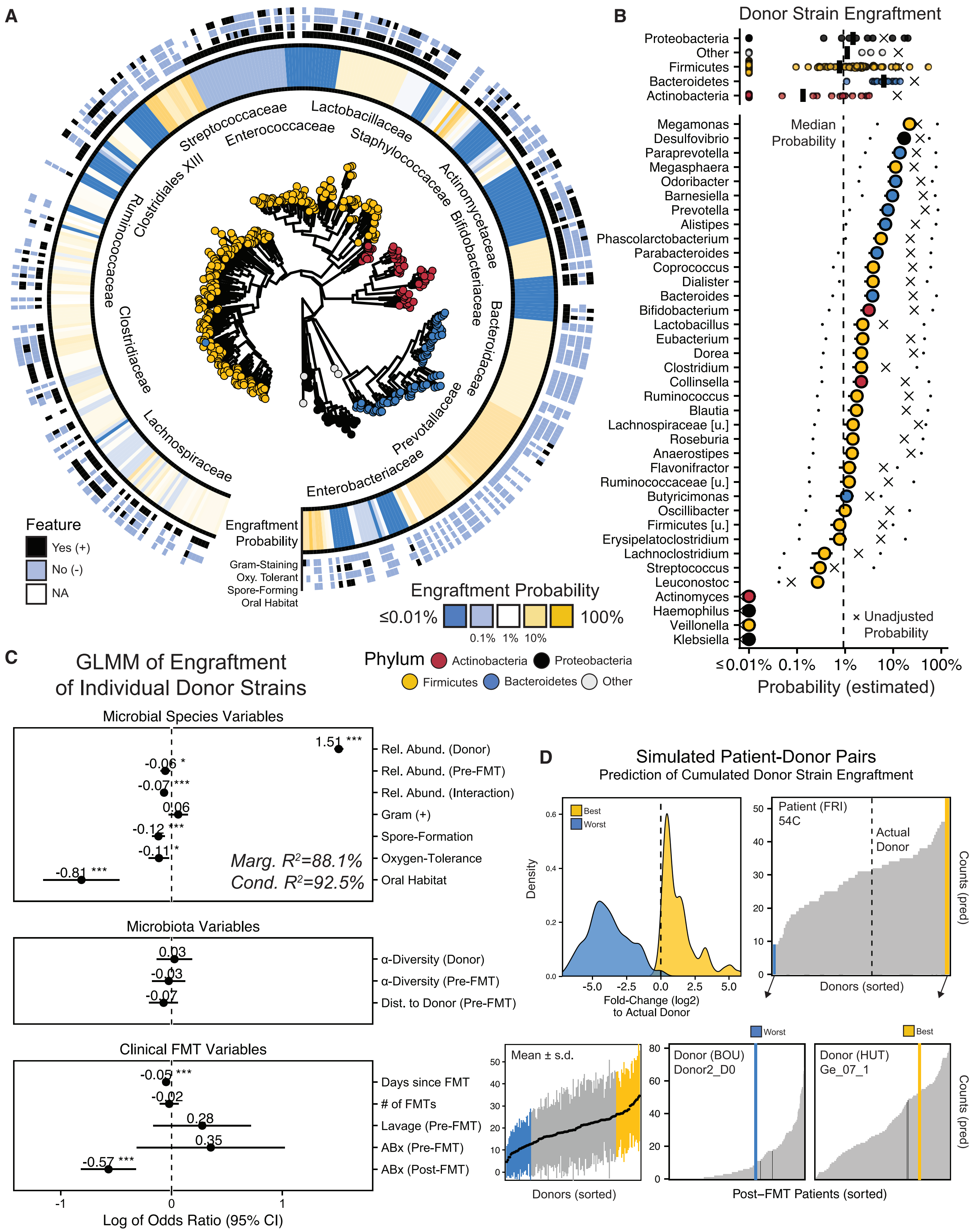

(legend on next page)

tolerance, as estimated using functional databases (see STAR Methods). Species properties were aggregated at the genus level to account for the inconsistent prevalence of species in different studies and donors and to provide us with a single, statistically robust, generalizable model to estimate donor strain engraftment.

Estimated donor strain engraftment probabilities (based on 83,351 species observations in donors) were generally higher for members of the phylum *Bacteroidetes* compared with *Firmicutes, Proteobacteria,* and *Actinobacteria* (Figure 5A). On the genus level, the median estimated engraftment probability was 0.92%, with *Megamonas* (21.7%), *Desulfovibrio* (16.8%), and *Paraprevotella* (13.7%) representing the most and *Klebsiella*, *Veillonella*, and *Haemophilus* (<2e-07%) the least likely engrafted genera (Figure 5B). In general, the GLMM-based estimated engraftment probabilities of individual species, adjusted for the influence of species, microbiota, and clinical variables, were substantially lower than their observed engraftment frequencies (see crosses in Figure 5B). Donor-derived *Bacteroides* strains, for example, engrafted in post-FMT patients at a relative frequency of 25.8%. However, *Bacteroides* strain engraftment was disproportionately more often observed in cases with FMT conditions that favored donor strain engraftment across all detected genera. As a consequence, the adjusted engraftment probability for *Bacteroides* strains, controlled for the effects of these conditions, was estimated at only 3.7%. Of the species variables, oral habitat (OR = −0.81, $p < 0.001$), spore formation (OR = −0.12, $p < 0.001$), and oxygen tolerance (OR = −0.11, $p < 0.05$) were all negatively correlated with engraftment probability (Figures 5C and Table S6). Across all detected microbial genera, donor strains were more likely to engraft if the corresponding species had a higher relative abundance in the donor (OR = 1.51; $p < 0.001$), but less likely to engraft if the corresponding species had a higher relative abundance in the recipient (OR = −0.06, $p < 0.05$) or if the species relative abundance was higher in the recipient relative to the donor (‘Interaction’, OR = −0.07, $p < 0.001$).

In conclusion, the engraftment of specific donor strains appears to be less dependent on microbiome (α/β-diversity) and clinical (ABx, lavage) parameters, than on associated species properties, such as (oral, spore-forming, oxygen-tolerant) lifestyle and (recipient and donor) relative abundance.

In silico prediction of donor strain engraftment for differentially abundant species and variable recipient/donor pairs

To explore the potential of FMT personalization, we assessed the effects of different, simulated donor species relative abundances and recipient/donor pairings on donor strain engraftment. First, donor strain engraftment probabilities were re-estimated for each FMT case based on the real input variable set, except that the species relative abundance in the donor was replaced with the highest or lowest species relative abundance that was detected in any of the other donors from the meta-cohort (Figure 5B). In this experimental setting, donor microbiota compositions were not qualitatively altered, i.e., only the relative abundance of the donor strain that was already present in the original sample was modified. Donor strain engraftment for many genera was predicted to become substantially more or less likely, in particular for genera from the phylum *Firmicutes* (Figure 5B). For example, while the genus *Ruminococcus* carried a median donor strain engraftment probability of 1.75% across the entire FMT meta-cohort, it was estimated that this probability could be reduced to 0.23% or increased to 58.9% with the highest and lowest detected cumulative relative abundance of *Ruminococcus* species in any of the other FMT donors, respectively. These simulations are noteworthy, as they suggest increased potential for the engraftment of specific strains from *in vitro*-supplemented donor samples.

Next, the total number of engrafted donor strains was re-estimated for all possible recipient/donor combinations from the meta-cohort. These predictions are informative, as the comparison of predicted and actual donor strain engraftment numbers in the subset of true recipient/donor combinations suggest a very good fit of our GLMM to the underlying data (r = 0.94, $p < 0.0001$). Across all simulated recipient/donor pairs, the predicted total number of engrafted donor strains per patient varied in a range of log2(±5)-fold between the worst and best-engrafting donors relative to the actual donor (Figure 5D). Donors that performed poorly in one recipient (<10 donor-derived post-FMT strains) were predicted to produce substantially larger numbers of engrafted strains in other patients (>50 donor-derived post-FMT strains) or vice versa (examples shown in Figure 5D). In general, differences between recipient/donor pairs accounted for more variation in the predicted number of engrafted donor strains than differences between donors

Figure 5. Donor strain engraftment probabilities for individual strains

(A) GLMM-based estimated donor strain engraftment probabilities in relation to phylogeny and microbial species features (Gram-staining, spore formation, oxygen tolerance, oral habitat).

(B) Median donor strain engraftment probabilities for different phyla (top) and genera (bottom), together with the estimated minimum and maximum probabilities, when using the lowest and highest species relative abundances that were detected in the meta-cohort as alternative input variables to the model. Crosses denote the donor strain engraftment probabilities without GLMM adjustments for the estimated influence of microbial, microbiota, and clinical FMT variables (see Figure 5C).

(C) Species features (patient and donor), microbiota parameters, and clinical FMT variables with relevance for the engraftment of individual donor strains based on the GLMM. Bars denote 95% confidence intervals (Wald). Asterisks denote significance thresholds: *$p \leq 0.01$, **$p \leq 0.001$, ***$p \leq 0.0001$.

(D) Total numbers of donor-derived strains in post-FMT patients, based on GLMM predictions for individual donor strains, vary substantially depending on recipient/donor pairing. Top left: They deviate ± 5-fold (log2) for the worst (blue) and best (yellow) simulated donor pair, relative to the actual recipient/donor pairs. Bottom left: Variations between different recipient/donor pairs for one donor (y axis range) generally exceed variations between different donors (x axis range). Black dots and bars: mean values ±SD. Top right, bottom middle, and right: Using patient 54C as an example, the predicted engraftment varies between <10 and >40 strains for different donors, but the worst (blue) and best (yellow) donors for this patient are predicted to also result in a broad range of engrafted donor strains in pairings with other patients. Dark lines indicate observations from actual recipient/donor pairings.

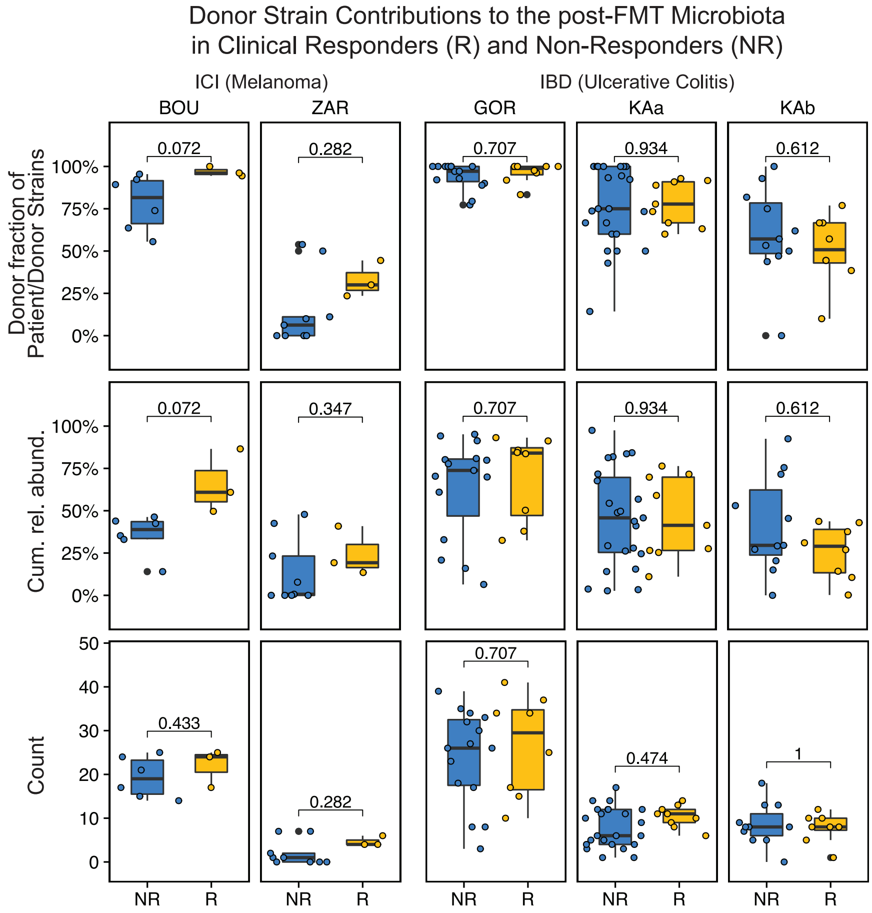

Figure 6. Donor microbiota engraftment and clinical response to FMT

Responders (R, yellow) and non-responders (NR, blue) from two FMT trials to overcome resistance to anti-PD-1 therapy in ICI-refractory melanoma patients and two FMT trials to induce remission in IBD patients were compared based on donor-derived strain fractions (of recipient and donor-derived strains), cumulative relative abundances of species represented by donor-derived strains and total numbers of donor-derived strains in post-FMT samples (last available sample within ≤ 4 months after FMT). See STAR Methods for a description of R/NR numbers and definitions. IBD patients from the two study branches of Paramsothy et al.[29], which applied different FMT protocols resulting in different levels of donor microbiota engraftment (Figure 3C), were compared separately; p-values based on the Wilcoxon test with false discovery rate (Benjamini-Hochberg) correction for multiple hypothesis testing within studies.

(Figure 5D), suggesting that recipient/donor matching may be more important to enhance donor strain engraftment than the use of "super-donors." Our simulations indicate potential for personalized FMT applications, based on recipient/donor pairings, which should be further clinically tested.

Strain engraftment may be linked to clinical response in rCDI and ICI but not IBD

Cure rates of ~90% after FMT have been reported for rCDI in patients after repeated antibiotic treatment failure,[9] which, based on our models, would induce dysbiosis in patients before and increase donor strain engraftment after FMT. In line with this expectation, rCDI patients from the meta-cohort exhibited large (>50%) and consistent donor-derived strain contributions to the post-FMT microbiota (Figure 3C). All rCDI patients resolved symptoms after the treatment, suggesting that the FMT outcome in this patient population may be dependent on donor microbiota engraftment, although the lack of fecal metagenomes from failed rCDI treatment cases prevented a systematic analysis of this relationship.

The FMT meta-cohort further included a subset of two ICI (BOU, ZAR) and two IBD (GOR, KAa/b) studies for which clinical response data were available (Figure 1A). Between these studies, donor strain engraftment rates varied considerably, in agreement with our predictions based on different clinical protocols used for antibiotic patient preparation and the number of applied FMTs (Figure 3C). These variations by far exceeded differences between responders and non-responders from the same study with respect to the number of donor-derived strains, their fraction of all detected recipient and donor-derived strains, or their cumulative relative abundance (Figure 6). Both ICI studies showed a trend toward larger contributions of donor-derived strains to the post-FMT microbiota of responders compared with non-responders. However, this trend was not significant (ZAR) or not significant after correcting for false discovery rate (BOU).

Between responders and non-responders from the two analyzed IBD studies, consistent differences regarding donor microbiota engraftment could neither be detected across nor within any of the studies or their subgroups (Figure 6). Further, none of the ICI or IBD studies showed a significant difference in ecological microbiome parameters, such as α/β-diversity or dysbiosis scores (Figure S8). The impact of donor microbiota engraftment on the clinical response to FMT therefore remains unclear and may be disease-dependent. The available data indicate that untargeted FMT with the goal to maximize donor microbiota engraftment may be more relevant for the treatment of rCDI than IBD or ICI-refractory melanoma, which may rather benefit from targeted FMT strategies with the goal to induce engraftment of specific donor species or strains.

DISCUSSION

FMT shows potential as a therapeutic tool for microbiome-associated pathologies and can be used as an experimental approach to establish causality for alterations of the human gut microbiota that have been associated with disease. However,

the relationship between donor microbiota engraftment and FMT outcomes in treated patients has not yet been comprehensively characterized, compared, and correlated with patient parameters, FMT modalities, and clinical outcomes. Moreover, the ecological principles that govern the post-FMT microbiota assembly process are poorly understood. Our analyses identify clinically modifiable factors as targets for FMT outcome optimization and differentiate between two distinct therapeutic goals: (1) to maximize broad, untargeted engraftment of the donor microbiota after FMT with antibiotic patient pretreatment, with likely clinical benefits for the treatment of rCDI; and (2) to increase the engraftment probability of specific donor strains by increasing the relative abundance of the corresponding species in the transplant sample or matching a recipient to an optimal donor. The second goal offers the prospect of developing personalized FMT applications for the treatment of different microbiota-associated pathologies in a framework of precision medicine.

A key finding of our study is that donor strain engraftment after FMT is strongly dependent on recipient microbiota composition and dysbiosis. In rCDI, where dysbiosis is rampant (Figure 1) and linked to taxonomic (e.g., lower α-diversity, altered β-diversity) and functional (e.g., increased relative abundance of oral and oxygen-tolerant species) microbiota disruption (Figure 2), FMT not only resolves dysbiosis but also results in contributions of 60%–90% donor strains to the post-FMT patient microbiota (Figure 3). In agreement with previous studies that did not detect strong dysbiotic microbiome patterns in IBD and obesity,[30–32] especially when controlling for confounding host variables,[33,34] we were unable to detect a major dysbiosis signal in other FMT-treated medical conditions, including IBD, MetS, and ICI. However, dysbiosis of the fecal microbiome was inducible by patient pretreatment with the broad-spectrum antibiotics (Figure 2). Similar to antibiotic treatment,[35] bowel lavage quantitatively reduces intestinal microbiota loads and alters fecal microbiota composition,[36] with consequences for colonization resistance to invading pathogens.[37] Accordingly, donor-derived strain contributions to the post-FMT microbiota were modest (<50%), unless patients underwent microbiota depletion by antibiotic treatment and bowel lavage, or received extensive repeated FMTs (>40 enemas) (Figure 3). The full therapeutic potential of FMT for the treatment of MetS patients therefore remains untested and unclear, as previous trials, which resulted in engraftment rates of <50% (Figure 3), were hampered by FMT protocols that lacked antibiotic patient microbiota depletion measures. By linking donor strain engraftment to quantifiable and pretreatment-dependent dysbiosis parameters in the patients before FMT, our findings can inform clinical practice on how to optimize FMT by increasing pre-FMT dysbiosis and selecting antibiotics for patient preparation. Importantly, as pre-FMT microbiota depletion induced extensive donor microbiota engraftment in all patient populations from the meta-cohort, our findings suggest that most microbiota-associated pathologies should be amenable to FMT-based therapeutic intervention.

Ecosystems assemble through a combination of deterministic, neutral, and historical processes.[38] Determining the relative importance of these processes for the microbial communities of the human gut will help us model, predict, and modulate microbiome assembly after FMT. By determining the ecological and clinical factors that shape microbiome assembly after FMT, our analyses can provide valuable mechanistic insights into the post-FMT microbiome assembly process. A positive association of donor species relative abundance and the number of applied FMTs with donor strain engraftment suggests an important role of propagule pressure for microbiome reorganization.[12] The concept of propagule pressure is used in invasion ecology to explain the successful colonization of a newly introduced population as determined by the quality, quantity, and frequency of invading organisms[39] identified as an important neutral or stochastic factor that shapes the post-FMT microbiome assembly process.[15] However, our finding that strains from predicted oral, oxygen-tolerant, and gram-positive species had a reduced chance of engraftment indicates that microbial adaptation to the gut environment is another relevant determinant of engraftment success. Microbiome assembly after FMT might thus be governed by resource competition and niche processes, which are deterministic and not neutral, and have recently been reported to control microbial colonization and resilience in post-FMT rCDI patients.[16]

Findings from our strain-level analysis provide insights into the ecological principles that shape the post-FMT patient microbiome, as well as the influence of clinical factors on these principles, specifically as they relate to (1) the conspecific coexistence of strains, and (2) the ratio by which donor strains engraft at the expense of recipient strains. In a community that is shaped by deterministic, niche-specific processes, such as the competition between members based on fitness differences, coexistence theory would postulate that closely related taxa with resource niche overlaps, such as conspecific strains, are more likely to compete than to coexist.[40] Priority effects, involving both ecological (niche preemption) and evolutionary (*in situ* evolution or genetic adaptation to the host environment) mechanisms,[41] would benefit earlier microbiome colonizers over later immigrating strains.[42,43] In our analysis, conspecific coexisting recipient and donor strains represented variable but small fractions of all detected strains in post-FMT patients (<10%, Figure 3C). While these findings are in agreement with those from a strain-level microbiota analysis of FMT-treated rCDI patients,[14] they contrast the 20%–53% reported for a metabolic syndrome patient cohort.[13] A recent preprint from the authors of the latter study described a reduced frequency of 22% conspecific coexisting strain observations in a more diverse post-FMT patient cohort, after detection of the same species in recipients and donors before FMT,[15] which is still considerably higher than the 2% conspecific coexistence rate observed in our analysis. However, microbial strains have been defined based on different biological, genomic, and bioinformatic concepts,[44] and while our strain profiling relies on the mapping of metagenomic reads to species-specific marker gene combinations to detect strain-specific single nucleotide variant (SNV) profiles,[22] Schmidt et al. used a combination of strain population-specific gene content and SNV profiles that demanded substantially fewer alignment sites for pairwise comparisons,[15] which may lead our method to underestimate and the latter to overestimate shared strain numbers.

Although we cannot rule out the possibility that a reduced sensitivity of our bioinformatic analysis toward low-abundant

strains is responsible for the decreased detection of strain coexistence, our findings are in agreement with strong niche-related competition and priority effects favoring recipient strains in undisturbed communities. Depletion of the resident microbiota is necessary to remove recipient strains, free ecological niches, and effectively overcome priority effects, resulting in increased donor strain engraftment in rCDI patients or recipients pretreated with antibiotics and bowel cleansing. The ability to overcome priority effects through antibiotic treatment has recently been demonstrated for consecutive strain colonization experiments in gnotobiotic mice.[45] Our findings support the importance of deterministic processes for post-FMT microbiome assembly, specifically the competition between closely related recipient and donor strains. Priority effects favor recipient strains, but their fitness advantage can be obliterated by antibiotics. Our ability to generate GLMMs that fit the heterogeneous meta-cohort data and model post-FMT microbiome assembly across all studies and pathologies further supports the relevance of deterministic factors for gut microbiome organization, as well as our conceptual ecological framework. This framework has implications for the personalization of FMTs and the development of defined probiotic consortia (mixtures of strains), which should be selected for maximum fitness and activity if used in the undisturbed setting of non-dysbiotic individuals.

Previous microbiome studies mostly lacked the taxonomic resolution to assess the role of donor strain engraftment for the clinical response to FMT in patients with microbiota-associated immunological or metabolic diseases, such as IBD and ICI. We compared donor strain engraftment in responders and non-responders from four IBD and ICI studies and did not observe differences in donor strain engraftment in ICI and IBD responders and non-responders. Moreover, although donor engraftment rates substantially varied between studies (Figure 6), clinical outcomes were remarkably similar, with reported clinical benefits after FMT in 40%[7] and 30%[8] of ICI patients, and clinical remission in 27%[29] and 24%[25] of IBD patients. However, association analyses between clinical response and donor microbiota engraftment in ICI patients were hindered by low patient numbers in both studies ($n \leq 15$), and ICI responders showed a consistent trend toward larger donor-derived strain fractions after FMT. Additional, larger strain-level microbiota analyses will be needed to detect and quantify the engraftment of specific donor strains and the replacement of recipient strains with genotypically different donor strains after FMT in relation to clinical benefits for IBD and ICI patients. Such donor strains would present attractive targets for follow-up experiments, such as FMT with supplemented samples, to prove a causal involvement in disease etiology[11] or refine FMT-based therapies.

Studies are beginning to outline personalized FMT strategies by drawing attention to both recipient and donor microbiome features to predict and optimize clinical FMT outcomes.[46] Our post-FMT microbiota assembly models estimated substantial variations (>10-fold) in the number of engrafted donor strains depending on the pairing of specific recipients with different donors from the meta-cohort. In addition, our models can be applied to predict engraftment probabilities for specific donor strains, providing a theoretical basis for precision microbiota modulation, by targeting specific donor strains with desirable genetic traits for introduction into a patient's microbiota, e.g., antibiotic-producing *Blautia producta* strains that inhibit vancomycin-resistant enterococci.[47] Our FMT outcome predictions suggest that testable strategies for the personalization of FMT should involve the supplementation of fecal samples with specific target strain cultures, as well as the selection of fecal samples from donor stool banks for recipient/donor matching.

In summary, our findings characterize the contribution of donor-derived strains to the post-FMT patient microbiota across a diverse set of patient, microbiome, and clinical conditions. They suggest a major impact of adjustable patient pretreatment modalities on donor strain engraftment and present generalizable predictive models for patient and strain-specific donor microbiota engraftment that are in agreement with ecological theory. They further illustrate the theoretical potential for personalized FMT applications through fecal supplementation with select strains and recipient/donor matching to increase the engraftment probability of specific strains. With this work, we lay the groundwork for future developments of precision microbiota modulation therapies.

Limitations of the study

Most clinical trials that were included in the analysis involved small patient numbers, which were sampled at different and inconsistent time points. Similarly, the control dataset of fecal metagenomes from healthy individuals was significantly larger than the patient or donor datasets from the FMT meta-cohort. Although our models identified strong and distinctive associations, these were partially based on study and patient subsets from the meta-cohort, and the temporal trajectories of the post-FMT assembly process were not characterized in detail. However, the use of a heterogeneous, but extensive, meta-cohort was also instrumental in the development of robust predictive generalized linear mixed models and the identification of what appear to be the universal drivers of post-FMT microbiota assembly. Our predictions for donor strain engraftment in other recipient/donor combinations or with fecal samples supplemented with bacterial cultures should be experimentally tested in follow-up clinical trials, as our retrospective study design did not allow for independent validation of our simulated FMT outcomes. Contributions of non-bacterial microbes, such as fungi and viruses/phages, to the post-FMT microbiome assembly, which may be clinically relevant,[48,49] as well as the impact of different antibiotic regimens on overall patient microbiota displacement and the engraftment of specific donor strains, should also be investigated. Quantitative information about absolute fecal microbiota abundance and the microbial density of the processed samples was not available, but will be needed to determine the impact of variable fecal bacterial densities in patients and donors on the detection of oral species and donor-derived strain fractions in post-FMT patients with different disease backgrounds. Finally, fecal microbiome analysis is generally unable to resolve the biogeography of microbial population dynamics along the gastrointestinal tract and distinguish, for example, between small and large intestinal donor strain engraftment that would exert different influences on the host and microbiota-associated diseases.

STAR★METHODS

Detailed methods are provided in the online version of this paper and include the following:

- KEY RESOURCES TABLE
- RESOURCE AVAILABILITY
  - Lead contact
  - Materials availability
  - Data and code availability
- EXPERIMENTAL MODEL AND SUBJECT DETAILS
  - Study design
  - Study cohort
  - Reference cohort
- METHOD DETAILS
  - Quality control and preprocessing of metagenomic shotgun sequence data
  - Taxonomic and functional microbiome profiling
  - Detection of shared strains with SameStr
- QUANTIFICATION AND STATISTICAL ANALYSIS
  - Analysis of taxonomic and functional community composition
  - Identification of recipient and donor-derived strains in post-FMT patients
  - Post-FMT microbiota assembly models
- ADDITIONAL RESOURCES

SUPPLEMENTAL INFORMATION

Supplemental information can be found online at https://doi.org/10.1016/j.xcrm.2022.100711.

ACKNOWLEDGMENTS

D.P. and W.F.F. received funding from the German Research Foundation (DFG, Project no. 316130265). M.D. and G.G. received funding from the Austrian Science fund (FWF, DK-MOLIN W1241). J.W. is funded in part by a Science Foundation Ireland Centre grant (APC/SFI/12/RC/2273_P2) and a Science Foundation Ireland Professorship (19/RP/6853).

AUTHOR CONTRIBUTIONS

Conceptualization and methodology, D.P., J.W., and W.F.F.; writing of the original draft, D.P., J.W., and W.F.F.; review & editing, D.P., J.W., and W.F.F.; software, validation, and formal analysis, D.P.; resources, M.D., S.P., N.O.K., and G.G.; data curation, D.P., M.D., S.P.; funding acquisition, W.F.F.

DECLARATION OF INTERESTS

The authors declare no competing interests.

Received: December 16, 2021
Revised: April 27, 2022
Accepted: July 14, 2022
Published: August 4, 2022

REFERENCES

1. D'Haens, G.R., and Jobin, C. (2019). Fecal microbial transplantation for diseases beyond recurrent Clostridium difficile infection. Gastroenterology *157*, 624–636.
2. Danne, C., Rolhion, N., and Sokol, H. (2021). Recipient factors in faecal microbiota transplantation: one stool does not fit all. Nat. Rev. Gastroenterol. Hepatol. *18*, 503–513.
3. Hooks, K.B., and O'Malley, M.A. (2017). Dysbiosis and its discontents. mBio *8*. e01492-17.
4. Kao, D., Roach, B., Silva, M., Beck, P., Rioux, K., Kaplan, G.G., Chang, H.-J., Coward, S., Goodman, K.J., Xu, H., et al. (2017). Effect of oral capsule–vs colonoscopy-delivered fecal microbiota transplantation on recurrent Clostridium difficile infection. JAMA *318*, 1985–1993.
5. van Nood, E., Vrieze, A., Nieuwdorp, M., Fuentes, S., Zoetendal, E.G., de Vos, W.M., Visser, C.E., Kuijper, E.J., Bartelsman, J.F.W.M., Tijssen, J.G.P., et al. (2013). Duodenal infusion of donor feces for recurrent Clostridium difficile. N. Engl. J. Med. *368*, 407–415.
6. Hanssen, N.M.J., de Vos, W.M., and Nieuwdorp, M. (2021). Fecal microbiota transplantation in human metabolic diseases: from a murky past to a bright future? Cell Metab. *33*, 1098–1110.
7. Davar, D., Dzutsev, A.K., McCulloch, J.A., Rodrigues, R.R., Chauvin, J.-M., Morrison, R.M., Deblasio, R.N., Menna, C., Ding, Q., Pagliano, O., et al. (2021). Fecal microbiota transplant overcomes resistance to anti-PD-1 therapy in melanoma patients. Science *371*, 595–602.
8. Baruch, E.N., Youngster, I., Ben-Betzalel, G., Ortenberg, R., Lahat, A., Katz, L., Adler, K., Dick-Necula, D., Raskin, S., Bloch, N., et al. (2021). Fecal microbiota transplant promotes response in immunotherapy-refractory melanoma patients. Science *371*, 602–609.
9. Khoruts, A., Staley, C., and Sadowsky, M.J. (2021). Faecal microbiota transplantation for Clostridioides difficile: mechanisms and pharmacology. Nat. Rev. Gastroenterol. Hepatol. *18*, 67–80.
10. Haifer, C., Paramsothy, S., Borody, T.J., Clancy, A., Leong, R.W., and Kaakoush, N.O. (2021). Long-term bacterial and fungal dynamics following oral lyophilized fecal microbiota transplantation in Clostridioides difficile infection. mSystems *6*. e00905-20.
11. Walter, J., Armet, A.M., Finlay, B.B., and Shanahan, F. (2020). Establishing or exaggerating causality for the gut microbiome: lessons from human microbiota-associated rodents. Cell *180*, 221–232.
12. Walter, J., Maldonado-Gómez, M.X., and Martínez, I. (2018). To engraft or not to engraft: an ecological framework for gut microbiome modulation with live microbes. Curr. Opin. Biotechnol. *49*, 129–139.
13. Li, S.S., Zhu, A., Benes, V., Costea, P.I., Hercog, R., Hildebrand, F., Huerta-Cepas, J., Nieuwdorp, M., Salojärvi, J., Voigt, A.Y., et al. (2016). Durable coexistence of donor and recipient strains after fecal microbiota transplantation. Science *352*, 586–589.
14. Smillie, C.S., Sauk, J., Gevers, D., Friedman, J., Sung, J., Youngster, I., Hohmann, E.L., Staley, C., Khoruts, A., Sadowsky, M.J., et al. (2018). Strain tracking reveals the determinants of bacterial engraftment in the human gut following fecal microbiota transplantation. Cell Host Microbe *23*, 229–240.e5.
15. Schmidt, T.S.B., Li, S.S., Maistrenko, O.M., Akanni, W., Coelho, L.P., Dolai, S., Fullam, A., Glazek, A.M., Hercog, R., Herrema, H., et al. (2021). Drivers and determinants of strain dynamics following faecal Microbiota transplantation. Preprint at bioRxiv. https://doi.org/10.1101/2021.09.30.462010.
16. Watson, A.R., Fuessel, J., Veseli, I., DeLongchamp, J.Z., Silva, M., Trigodet, F., Lolans, K., Shaiber, A., Fogarty, E., Runde, J.M., et al. (2021). Adaptive ecological processes and metabolic independence drive microbial colonization and resilience in the human gut. Preprint at bioRxiv. https://doi.org/10.1101/2021.03.02.433653.
17. Xiao, Y., Angulo, M.T., Lao, S., Weiss, S.T., and Liu, Y.-Y. (2020). An ecological framework to understand the efficacy of fecal microbiota transplantation. Nat. Commun. *11*, 3329.
18. Lawley, T.D., and Walker, A.W. (2013). Intestinal colonization resistance. Immunology *138*, 1–11.
19. Stecher, B., and Hardt, W.-D. (2008). The role of microbiota in infectious disease. Trends Microbiol. *16*, 107–114.

20. Battaglioli, E.J., Hale, V.L., Chen, J., Jeraldo, P., Ruiz-Mojica, C., Schmidt, B.A., Rekdal, V.M., Till, L.M., Huq, L., Smits, S.A., et al. (2018). Uses amino acids associated with gut microbial dysbiosis in a subset of patients with diarrhea. Sci. Transl. Med. *10*, eaam7019.

21. Song, Y., Garg, S., Girotra, M., Maddox, C., von Rosenvinge, E.C., Dutta, A., Dutta, S., and Fricke, W.F. (2013). Microbiota dynamics in patients treated with fecal microbiota transplantation for recurrent Clostridium difficile infection. PLoS One *8*, e81330.

22. Podlesny, D., Arze, C., Dörner, E., Verma, S., Dutta, S., Walter, J., and Fricke, W.F. (2022). Metagenomic strain detection with SameStr: identification of a persisting core gut microbiota transferable by fecal transplantation. Microbiome *10*, 53.

23. Gevers, D., Kugathasan, S., Denson, L.A., Vázquez-Baeza, Y., Van Treuren, W., Ren, B., Schwager, E., Knights, D., Song, S.J., Yassour, M., et al. (2014). The treatment-naive microbiome in new-onset Crohn's disease. Cell Host Microbe *15*, 382–392.

24. Leffler, D.A., and Lamont, J.T. (2015). Clostridium difficile infection. N. Engl. J. Med. *372*, 1539–1548.

25. Kump, P., Wurm, P., Gröchenig, H.P., Wenzl, H., Petritsch, W., Halwachs, B., Wagner, M., Stadlbauer, V., Eherer, A., Hoffmann, K.M., et al. (2018). The taxonomic composition of the donor intestinal microbiota is a major factor influencing the efficacy of faecal microbiota transplantation in therapy refractory ulcerative colitis. Aliment. Pharmacol. Ther. *47*, 67–77.

26. Bar-Yoseph, H., Carasso, S., Shklar, S., Korytny, A., Even Dar, R., Daoud, H., Nassar, R., Maharshak, N., Hussein, K., Geffen, Y., et al. (2020). Oral capsulized Fecal microbiota transplantation for eradication of carbapenemase-producing Enterobacteriaceae colonization with a metagenomic perspective. Clin. Infect. Dis. *73*, e166–e175.

27. Leo, S., Lazarevic, V., Girard, M., Gaïa, N., Schrenzel, J., de Lastours, V., Fantin, B., Bonten, M., Carmeli, Y., Rondinaud, E., et al. (2020). Metagenomic characterization of gut microbiota of carriers of extended-spectrum beta-lactamase or carbapenemase-producing Enterobacteriaceae following treatment with oral antibiotics and fecal microbiota transplantation: results from a multicenter randomized trial. Microorganisms *8*, E941.

28. Podlesny, D., and Fricke, W.F. (2021). Strain inheritance and neonatal gut microbiota development: a meta-analysis. Int. J. Med. Microbiol. *311*, 151483.

29. Paramsothy, S., Nielsen, S., Kamm, M.A., Deshpande, N.P., Faith, J.J., Clemente, J.C., Paramsothy, R., Walsh, A.J., van den Bogaerde, J., Samuel, D., et al. (2019). Specific bacteria and metabolites associated with response to fecal microbiota transplantation in patients with ulcerative colitis. Gastroenterology *156*, 1440–1454.e2.

30. Lloyd-Price, J., Arze, C., Ananthakrishnan, A.N., Schirmer, M., Avila-Pacheco, J., Poon, T.W., Andrews, E., Ajami, N.J., Bonham, K.S., Brislawn, C.J., et al. (2019). Multi-omics of the gut microbial ecosystem in inflammatory bowel diseases. Nature *569*, 655–662.

31. Duvallet, C., Gibbons, S.M., Gurry, T., Irizarry, R.A., and Alm, E.J. (2017). Meta-analysis of gut microbiome studies identifies disease-specific and shared responses. Nat. Commun. *8*, 1784.

32. Sze, M.A., and Schloss, P.D. (2016). Looking for a signal in the noise: revisiting obesity and the microbiome. mBio *7*. e01018-16.

33. Vujkovic-Cvijin, I., Sklar, J., Jiang, L., Natarajan, L., Knight, R., and Belkaid, Y. (2020). Host variables confound gut microbiota studies of human disease. Nature *587*, 448–454.

34. Stanislawski, M.A., Dabelea, D., Lange, L.A., Wagner, B.D., and Lozupone, C.A. (2019). Gut microbiota phenotypes of obesity. NPJ Biofilms Microbiomes *5*, 18.

35. Modi, S.R., Collins, J.J., and Relman, D.A. (2014). Antibiotics and the gut microbiota. J. Clin. Invest. *124*, 4212–4218.

36. Jalanka, J., Salonen, A., Salojärvi, J., Ritari, J., Immonen, O., Marciani, L., Gowland, P., Hoad, C., Garsed, K., Lam, C., et al. (2015). Effects of bowel cleansing on the intestinal microbiota. Gut *64*, 1562–1568.

37. Litvak, Y., and Bäumler, A.J. (2019). The founder hypothesis: a basis for microbiota resistance, diversity in taxa carriage, and colonization resistance against pathogens. PLoS Pathog. *15*, e1007563.

38. Cavender-Bares, J., Kozak, K.H., Fine, P.V.A., and Kembel, S.W. (2009). The merging of community ecology and phylogenetic biology. Ecol. Lett. *12*, 693–715.

39. Lockwood, J.L., Cassey, P., and Blackburn, T. (2005). The role of propagule pressure in explaining species invasions. Trends Ecol. Evol. *20*, 223–228.

40. Letten, A.D., Ke, P., and Fukami, T. (2017). Linking modern coexistence theory and contemporary niche theory. Ecol. Monogr. *87*, 161–177.

41. De Meester, L., Vanoverbeke, J., Kilsdonk, L.J., and Urban, M.C. (2016). Evolving perspectives on monopolization and priority effects. Trends Ecol. Evol. *31*, 136–146.

42. Fukami, T. (2015). Historical contingency in community assembly: integrating niches, species pools, and priority effects. Annu. Rev. Ecol. Evol. Syst. *46*, 1–23.

43. Martínez, I., Maldonado-Gomez, M.X., Gomes-Neto, J.C., Kittana, H., Ding, H., Schmaltz, R., Joglekar, P., Cardona, R.J., Marsteller, N.L., Kembel, S.W., et al. (2018). Experimental evaluation of the importance of colonization history in early-life gut microbiota assembly. Elife *7*, e36521.

44. Van Rossum, T., Ferretti, P., Maistrenko, O.M., and Bork, P. (2020). Diversity within species: interpreting strains in microbiomes. Nat. Rev. Microbiol. *18*, 491–506.

45. Segura Munoz, R.R., Mantz, S., Martínez, I., Li, F., Schmaltz, R.J., Pudlo, N.A., Urs, K., Martens, E.C., Walter, J., and Ramer-Tait, A.E. (2022). Experimental evaluation of ecological principles to understand and modulate the outcome of bacterial strain competition in gut microbiomes. ISME J. *16*, 1594–1604.

46. Benech, N., and Sokol, H. (2020). Fecal microbiota transplantation in gastrointestinal disorders: time for precision medicine. Genome Med. *12*, 58.

47. Kim, S.G., Becattini, S., Moody, T.U., Shliaha, P.V., Littmann, E.R., Seok, R., Gjonbalaj, M., Eaton, V., Fontana, E., Amoretti, L., et al. (2019). Microbiota-derived lantibiotic restores resistance against vancomycin-resistant Enterococcus. Nature *572*, 665–669.

48. Leonardi, I., Paramsothy, S., Doron, I., Semon, A., Kaakoush, N.O., Clemente, J.C., Faith, J.J., Borody, T.J., Mitchell, H.M., Colombel, J.-F., et al. (2020). Fungal trans-kingdom dynamics linked to responsiveness to fecal microbiota transplantation (FMT) therapy in ulcerative colitis. Cell Host Microbe *27*, 823–829.e3.

49. Manrique, P., Zhu, Y., van der Oost, J., Herrema, H., Nieuwdorp, M., de Vos, W.M., and Young, M. (2021). Gut bacteriophage dynamics during fecal microbial transplantation in subjects with metabolic syndrome. Gut Microb. *13*, 1–15.

50. Ng, S.C., Xu, Z., Mak, J.W.Y., Yang, K., Liu, Q., Zuo, T., Tang, W., Lau, L., Lui, R.N., Wong, S.H., et al. (2021). Microbiota engraftment after faecal microbiota transplantation in obese subjects with type 2 diabetes: a 24-week, double-blind, randomised controlled trial. Gut *71*, 716–723.

51. Wilson, B.C., Vatanen, T., Jayasinghe, T.N., Leong, K.S.W., Derraik, J.G.B., Albert, B.B., Chiavaroli, V., Svirskis, D.M., Beck, K.L., Conlon, C.A., et al. (2021). Strain engraftment competition and functional augmentation in a multi-donor fecal microbiota transplantation trial for obesity. Microbiome *9*, 107.

52. Kong, L., Lloyd-Price, J., Vatanen, T., Seksik, P., Beaugerie, L., Simon, T., Vlamakis, H., Sokol, H., and Xavier, R.J. (2020). Linking strain engraftment in fecal microbiota transplantation with maintenance of remission in Crohn's disease. Gastroenterology *159*, 2193–2202.e5.

53. Seymour, L., Bogaerts, J., Perrone, A., Ford, R., Schwartz, L.H., Mandrekar, S., Lin, N.U., Litière, S., Dancey, J., Chen, A., et al. (2017). iRECIST: guidelines for response criteria for use in trials testing immunotherapeutics. Lancet Oncol. *18*, e143–e152.

54. Eisenhauer, E.A., Therasse, P., Bogaerts, J., Schwartz, L.H., Sargent, D., Ford, R., Dancey, J., Arbuck, S., Gwyther, S., Mooney, M., et al. (2009). New response evaluation criteria in solid tumours: revised RECIST guideline (version 1.1). Eur. J. Cancer *45*, 228–247.

55. Lewis, J.D., Chuai, S., Nessel, L., Lichtenstein, G.R., Aberra, F.N., and Ellenberg, J.H. (2008). Use of the noninvasive components of the Mayo score to assess clinical response in ulcerative colitis. Inflamm. Bowel Dis. *14*, 1660–1666.

56. Pasolli, E., Schiffer, L., Manghi, P., Renson, A., Obenchain, V., Truong, D.T., Beghini, F., Malik, F., Ramos, M., Dowd, J.B., et al. (2017). Accessible, curated metagenomic data through ExperimentHub. Nat. Methods *14*, 1023–1024.

57. Beghini, F., McIver, L.J., Blanco-Míguez, A., Dubois, L., Asnicar, F., Maharjan, S., Mailyan, A., Manghi, P., Scholz, M., Thomas, A.M., et al. (2021). Integrating taxonomic, functional, and strain-level profiling of diverse microbial communities with bioBakery 3. Elife *10*, e65088.

58. Human Microbiome Project Consortium (2012). Structure, function and diversity of the healthy human microbiome. Nature *486*, 207–214.

59. Raymond, F., Ouameur, A.A., Déraspe, M., Iqbal, N., Gingras, H., Dridi, B., Leprohon, P., Plante, P.-L., Giroux, R., Bérubé, È., et al. (2016). The initial state of the human gut microbiome determines its reshaping by antibiotics. ISME J. *10*, 707–720.

60. Zeller, G., Tap, J., Voigt, A.Y., Sunagawa, S., Kultima, J.R., Costea, P.I., Amiot, A., Böhm, J., Brunetti, F., Habermann, N., et al. (2014). Potential of fecal microbiota for early-stage detection of colorectal cancer. Mol. Syst. Biol. *10*, 766.

61. Yu, J., Feng, Q., Wong, S.H., Zhang, D., Liang, Q.Y., Qin, Y., Tang, L., Zhao, H., Stenvang, J., Li, Y., et al. (2017). Metagenomic analysis of faecal microbiome as a tool towards targeted non-invasive biomarkers for colorectal cancer. Gut *66*, 70–78.

62. Feng, Q., Liang, S., Jia, H., Stadlmayr, A., Tang, L., Lan, Z., Zhang, D., Xia, H., Xu, X., Jie, Z., et al. (2015). Gut microbiome development along the colorectal adenoma-carcinoma sequence. Nat. Commun. *6*, 6528.

63. Vogtmann, E., Hua, X., Zeller, G., Sunagawa, S., Voigt, A.Y., Hercog, R., Goedert, J.J., Shi, J., Bork, P., and Sinha, R. (2016). Colorectal cancer and the human gut microbiome: reproducibility with whole-genome shotgun sequencing. PLoS One *11*, e0155362.

64. Thomas, A.M., Manghi, P., Asnicar, F., Pasolli, E., Armanini, F., Zolfo, M., Beghini, F., Manara, S., Karcher, N., Pozzi, C., et al. (2019). Metagenomic analysis of colorectal cancer datasets identifies cross-cohort microbial diagnostic signatures and a link with choline degradation. Nat. Med. *25*, 667–678.

65. Wirbel, J., Pyl, P.T., Kartal, E., Zych, K., Kashani, A., Milanese, A., Fleck, J.S., Voigt, A.Y., Palleja, A., Ponnudurai, R., et al. (2019). Meta-analysis of fecal metagenomes reveals global microbial signatures that are specific for colorectal cancer. Nat. Med. *25*, 679–689.

66. Yachida, S., Mizutani, S., Shiroma, H., Shiba, S., Nakajima, T., Sakamoto, T., Watanabe, H., Masuda, K., Nishimoto, Y., Kubo, M., et al. (2019). Metagenomic and metabolomic analyses reveal distinct stage-specific phenotypes of the gut microbiota in colorectal cancer. Nat. Med. *25*, 968–976.

67. Langmead, B., and Salzberg, S.L. (2012). Fast gapped-read alignment with Bowtie 2. Nat. Methods *9*, 357–359.

68. Lê, S., Josse, J., and Husson, F. (2008). FactoMineR: AnRPackage for multivariate analysis. J. Stat. Softw. *25*. https://doi.org/10.18637/jss.v025.i01.

69. Wilke, C.O. (2021). Ggridges: Ridgeline Plots in "Ggplot2.

70. R Core Team (2019). R: A Language and Environment for Statistical Computing (R Core Team).

71. Oksanen, J., Blanchet, F.G., Friendly, M., Kindt, R., Legendre, P., McGlinn, D., Minchin, P.R., O'Hara, R.B., Simpson, G.L., Solymos, P., et al. (2020). Vegan: Community Ecology Package.

72. McMurdie, P.J., and Holmes, S. (2013). phyloseq: an R package for reproducible interactive analysis and graphics of microbiome census data. PLoS One *8*, e61217.

73. Browne, H.P., Forster, S.C., Anonye, B.O., Kumar, N., Neville, B.A., Stares, M.D., Goulding, D., and Lawley, T.D. (2016). Culturing of "unculturable" human microbiota reveals novel taxa and extensive sporulation. Nature *533*, 543–546.

74. Vatanen, T., Plichta, D.R., Somani, J., Münch, P.C., Arthur, T.D., Hall, A.B., Rudolf, S., Oakeley, E.J., Ke, X., Young, R.A., et al. (2019). Genomic variation and strain-specific functional adaptation in the human gut microbiome during early life. Nat. Microbiol. *4*, 470–479.

75. Reimer, L.C., Vetcininova, A., Carbasse, J.S., Söhngen, C., Gleim, D., Ebeling, C., and Overmann, J. (2019). BacDive in 2019: bacterial phenotypic data for High-throughput biodiversity analysis. Nucleic Acids Res. *47*, D631–D636.

76. Zhu, C., Mahlich, Y., Miller, M., and Bromberg, Y. (2018). fusionDB: assessing microbial diversity and environmental preferences via functional similarity networks. Nucleic Acids Res. *46*, D1283–D1541.

77. Sierra, M.A., Bhattacharya, C., Ryon, K., Meierovich, S., Shaaban, H., Westfall, D., Mohammad, R., Kuchin, K., Afshinnekoo, E., Danko, D.C., et al. (2019). The Microbe Directory v2.0: an expanded database of ecological and phenotypical features of microbes. Preprint at bioRxiv. https://doi.org/10.1101/2019.12.20.860569.

78. Escapa, I.F., Chen, T., Huang, Y., Gajare, P., Dewhirst, F.E., and Lemon, K.P. (2018). New insights into human nostril microbiome from the expanded human oral microbiome database (eHOMD): a resource for the microbiome of the human aerodigestive tract. mSystems *3*. e00187-18.

79. Bates, D., Mächler, M., Bolker, B., and Walker, S. (2015). Fitting linear mixed-effects models Usinglme4. J. Stat. Softw. *67*. https://doi.org/10.18637/jss.v067.i01.

80. Kuznetsova, A., Brockhoff, P.B., and Christensen, R.H.B. (2017). LmerTest package: tests in linear mixed effects models. J. Stat. Softw. *82*. https://doi.org/10.18637/jss.v082.i13.

81. Lüdecke, D., Ben-Shachar, M., Patil, I., Waggoner, P., and Makowski, D. (2021). Performance: an R package for assessment, comparison and testing of statistical models. J. Open Source Softw. *6*, 3139.

82. Lüdecke, D. (2018). Ggeffects: tidy data frames of marginal effects from regression models. J. Open Source Softw. *3*, 772.

83. Lüdecke, D. (2021). sjPlot: Data Visualization for Statistics in Social Science.

84. Chen, T., He, T., Benesty, M., Khotilovich, V., Tang, Y., Cho, H., Chen, K., Mitchell, R., Cano, I., Zhou, T., et al. (2021). Xgboost: Extreme Gradient Boosting.

85. Yu, G., Lam, T.T.-Y., Zhu, H., and Guan, Y. (2018). Two methods for mapping and visualizing associated data on phylogeny using ggtree. Mol. Biol. Evol. *35*, 3041–3043.

86. Yu, G., Smith, D.K., Zhu, H., Guan, Y., and Lam, T.T. (2017). ggtree: an R package for visualization and annotation of phylogenetic trees with their covariates and other associated data. Methods Ecol. Evol. *8*, 28–36.

CellPress
OPEN ACCESS

STAR★METHODS

KEY RESOURCES TABLE

REAGENT or RESOURCE	SOURCE	IDENTIFIER
Deposited data		
Newly generated raw WGS data for the Study Cohort (IBD, GOR)	Kump et al., 2018	PRJEB47061
Previously published raw WGS data for the Study Cohort	see Table S2	see Table S2 for ENA accession numbers
Previously published raw WGS data for the Reference Cohort	see Table S3	see Table S3 ENA accession numbers
Software and algorithms		
SameStr v1	Podlesny et al., 2022	https://github.com/danielpodlesny/SameStr
MetaPhlAn v3.0.7 with mpa_v30, 201901	Beghini et al., 2021	https://github.com/biobakery/MetaPhlAn
KneadData v0.6.1	Beghini et al., 2021	https://github.com/biobakery/kneaddata
Bowtie2 v2.2.3	Langmead and Salzberg, 2012	http://bowtie-bio.sourceforge.net/bowtie2/
R v3.6.1	R Core Team, 2019	https://www.r-project.org/
R package: curatedMetagenomicsData	Pasolli et al., 2017	https://doi.org/10.18129/B9.bioc.curatedMetagenomicData
R package: vegan v2.5.7	Oksanen et al., 2020	https://cran.r-project.org/web/packages/vegan/
R package: phyloseq v1.28.0	McMurdie and Holmes, 2013	https://doi.org/10.18129/B9.bioc.phyloseq
R package: lme4 v1.1.27	Bates et al., 2015	https://cran.r-project.org/web/packages/lme4/
R package: lmerTest v3.1.3	Kuznetsova et al., 2017	https://cran.r-project.org/web/packages/lmerTest/
R package: performance v0.7.2	Lüdecke et al., 2021	https://cran.r-project.org/web/packages/performance/
R package: ggeffects v1.1.0	Lüdecke, 2018	https://cran.r-project.org/web/packages/ggeffects/
R package: sjPlot v2.8.8	Lüdecke, 2021	https://cran.r-project.org/web/packages/sjPlot/
R package: xgboost v1.4.1.1	Chen et al., 2021	https://cran.r-project.org/web/packages/xgboost/
R package: ggtree v1.16.6	Yu et al., 2017a, 2018	https://doi.org/10.18129/B9.bioc.ggtree
R package: FactoMineR v2.4	Lê et al., 2008	https://cran.r-project.org/web/packages/FactoMineR/
R package: ggridges v0.5.3	Wilke, 2021	https://cran.r-project.org/web/packages/ggridges/

RESOURCE AVAILABILITY

Lead contact

Further information and requests for resources and reagents should be directed to and will be fulfilled by the lead contact, W. Florian Fricke (w.florian.fricke@uni-hohenheim.de).

Materials availability

This study did not generate new unique reagents.

Data and code availability

- Previously published and newly generated metagenomic sequence data are available from the European Nucleotide Archive under the accession numbers that are listed in the key resources table.
- This paper does not report original code.
- Any additional information required to reanalyze the data reported in this paper is available from the lead contact upon request.

EXPERIMENTAL MODEL AND SUBJECT DETAILS

Study design

The objective of this study was to characterize the microbiota of FMT-treated patients with different medical conditions ("meta-cohort") and their donors and to apply strain-level microbiota profiling in order to determine donor-derived strain contributions to

post-FMT microbiota assembly. This information was used to inform predictive models to describe the post-FMT microbiota assembly process and to determine the role of recipient and donor microbiota features, taxonomic and ecological microbiota parameters, and clinical modalities of patient preparation and FMT for donor strain engraftment.

Study cohort

We assembled a comprehensive meta-cohort of available metagenomics data from clinical FMT trials, including 254 distinct clinical cases in which FMT was used to modulate the microbiota of patients with different medical conditions (Figure 1A). Published across thirteen distinct studies, FMT was used to treat recurrent *Clostridioides difficile* infection (rCDI),[14,16,21] type 2 diabetes mellitus, obesity and metabolic syndrome (MetS),[13,50,51] the inflammatory bowel diseases Crohn's disease and ulcerative colitis (IBD),[25,29,52] to eradicate multidrug-resistant *Enterobacteriaceae* carriage (MDR),[26,27] and to induce response to anti–PD-1 immunotherapy in immune checkpoint inhibitor therapy-refractory melanoma patients (ICI).[7,8] Fecal metagenomic shotgun sequence data were included from a total of 1322 samples obtained from patients before and (often multiple times) after FMT (Figure S1A), as well as from stool donors (Table S1). Metadata were obtained from the supplementary information provided with each publication or from the authors.

Clinical response information was obtained from the original publications and defined as follows: For ICI patients (see Figure 1B in both Baruch et al. and Davar et al.), responders (R, BOU = 3, ZAR = 3) experienced an objective response to treatment as per Response Evaluation Criteria in Solid Tumors (BOU: iRECIST,[53] ZAR: RECIST v1.1[54]), indicated by a tumor regression of at least 30% compared to baseline, whereas non-responders (NR, BOU = 6, ZAR = 9) showed progressive disease with an increase in tumor size of at least 20%. For IBD patients, responders (R, GOR = 8, KAa = 9, KAb = 8) went into remission (Mayo score[55]: ≤ 2), all other IBD patients were classified as non-responders (NR, GOR = 15, KAa = 23, KAb = 11).

Reference cohort

To compare microbiota composition metrics against a healthy control cohort, fecal metagenomic shotgun sequence data from subjects that had not reported conditions that would suggest extensive medication or strong microbiota perturbations were obtained through the curatedMetagenomicsData package.[56] For each subject, sequence data downloaded from NCBI's Sequence Read Archive were concatenated in case of multiple available accessions. We additionally collected preprocessed MetaPhlAn3 species-level taxonomic relative abundance profiles that were made available with bioBakery 3.[57] In sum, 739 samples from nine publicly available datasets (Table S1) were used for the reference cohort.[58–66]

METHOD DETAILS

Quality control and preprocessing of metagenomic shotgun sequence data

All raw paired-end metagenomic sequence reads were processed with KneadData v0.6.1 to trim sequence regions with base call quality below Q20 within a 4-nucleotide sliding window and to remove reads that were truncated by more than 30% (SLIDINGWINDOW:4:20, MINLEN:70). To remove host contamination, trimmed reads were mapped against the human genome (GRCh37/hg19) with Bowtie2 v2.2.3.[67] Output files consisting of surviving paired and orphan reads were concatenated and used for further processing.

Taxonomic and functional microbiome profiling

Taxonomic analyses were carried out to provide an overview of sample microbiota compositions and to generate the marker gene alignments that served as input for the strain-level analysis with SameStr. Preprocessed sequence reads from each sample were mapped against the MetaPhlAn clade-specific marker gene database (mpa_v30, 201901, Table S2) using MetaPhlAn3 v3.0.7.[57] Relative abundances of species-level taxonomic profiles were centered-log ratio (clr) transformed and used for principal component analysis with FactoMineR v2.4.[68] Density ridgeline plots were generated with the ggridges package v0.5.3[69] in R v3.6.1.[70] Shannon Index and Bray-Curtis dissimilarity were determined with vegan v2.5.7 (diversity function[71]), and the UniFrac distance with phyloseq v1.28.0 (UniFrac function[72]) using the phylogenetic tree published along MetaPhlAn3. Functional metadata on bacterial species (Table S5) were aggregated from different publications,[73,74] the List of Prokaryotes according to their Aerotolerant or Obligate Anaerobic Metabolism (OXYTOL 1.3, Mediterranean institute of infection in Marseille), bacDive,[75] FusionDB,[76] The Microbe Directory v2.0,[77] and the expanded Human Oral Microbiome Database.[78] For each sample, the cumulative relative abundance of taxa that were associated with oxygen tolerance or an oral habitat was determined (Figure 2). The Microbial Dysbiosis Score was calculated as the log-ratio of the cumulative relative abundance of taxa which were previously positively and negatively associated with pediatric Crohn's Disease.[23]

Detection of shared strains with SameStr

To track bacterial strains in distinct biological samples, we used the SameStr tool from our group,[22] which leverages the clade-specific MetaPhlAn markers to resolve within-species phylogenetic sequence variations. Briefly, MetaPhlAn3 marker alignments were converted to single nucleotide variant (SNV) profiles, extensively filtered, merged, and compared between metagenomic samples based on the maximum variant profile similarity (MVS) to detect strains that were shared between samples. In contrast to

StrainPhlAn, which uses the major allele at every position in the alignment (consensus sequence), SameStr's MVS-based approach evaluates the co-occurrence of all four possible nucleotide alleles between overlapping alignment sites of two samples, including polymorphic sites ($\geq$10% allele frequency), which can result from multiple strains representing the same species. This allows for the detection of sub-dominant shared strains or coexisting recipient and donor strains from the same species. Shared strains were called if species alignments between metagenomic samples overlapped by $\geq$ 5 kb and with an MVS of $\geq$99.9%. Additional documentation of SameStr is available at https://www.github.com/danielpodlesny/SameStr.

QUANTIFICATION AND STATISTICAL ANALYSIS

Analysis of taxonomic and functional community composition

To detect significant differences in patients and donors relative to healthy controls (Figure 2) with respect to microbiota composition (α/β-diversity), dysbiosis and bacterial species lifestyles (oral habitat, oxygen tolerance) generalized linear mixed-effects models (GLMMs) were used, which were calculated with the glmer function (binomial distribution, logit link, 'Oxy. tolerant (%)', 'Oral habitat (%)', 'Spore forming (%)') in lme4 v1.1.27[79] or the lmer function (Gaussian distribution, 'Dysbiosis (Score)', 'Shannon Index', 'Dist. to Ctrls') from lmerTest v3.1.3.[80] For each study and metric, the sample type (pre-FMT^{ABx-}, pre-FMT^{ABx+}, post-FMT, donor, control) was incorporated as a fixed effect, using samples from the control cohort as a reference. Only post-FMT patient sample data were included that were collected at least five days after FMT. We controlled for study effects in the control cohort and repeated post-FMT patient sampling by including study and case as random effects. Multicollinearity was assessed by calculating the variance inflation factor with the performance package v0.7.2 (multicollinearity function[81]). The resulting model outputs are tabulated in Table S3.

Identification of recipient and donor-derived strains in post-FMT patients

For each post-FMT patient sample, recipient and donor-derived taxa were determined based on shared species and strains with pre-FMT recipient and donor samples. Recipient or donor-derived or coexisting strains were identified as shared exclusively between post-FMT and pre-FMT patient, or between post-FMT patient and donor, or between post-FMT and pre-FMT patient and donor samples, respectively. Analogously, recipient or donor-derived species were exclusively shared between post-FMT and pre-FMT patient, or between post-FMT patient and donor samples, respectively. In several cases, multiple available samples from the same donor or individual samples from multiple donors that were used for pooled FMTs were combined. For Wilson et al. (OSU), individual donor samples that the authors had combined in batches for FMT were combined. For Li et al. (BOR), which included data from a single donor sampled at three distinct time points without providing information about their specific use for distinct patients, donor samples were combined, as described in the original publication. For Kump et al. (GOR), which included repeated FMT, all available sequence data for each donor were combined across treatment rounds. Ng et al. (CHA) did not disclose sample metadata in the publication, including information about patient/donor pairing and patient assignments to different treatment groups (FMT alone, FMT with lifestyle intervention, or sham treatment), and this information was not made available upon requests to the authors. For this study, concatenated sequence files of all five donor samples were used and sham cases identified and excluded based on the lack of shared strains with any post-FMT sample (see Figure S5). In case of pooled donor sequence data, mean values of all relevant microbiota metrics (e.g. α/β-diversity) were used in our models. Baruch et al. (BOU) published fecal metagenomes from patients before but not after antibiotic pretreatment. To include this dataset in our models, we imputed pre-FMT microbiota metrics with the mean values that were observed in all antibiotically pretreated patients. We also tested our model without the BOU data, which had only a minor effect on the model predictions (Figure S5).

Post-FMT microbiota assembly models

Generalized linear mixed-effects models (GLMMs) were used to estimate the effects of recipient and donor microbiota parameters and clinical modalities on the post-FMT microbiota assembly process. Separate GLMMs were used to determine the role of these parameters for donor-derived strain fractions in individual patients after FMT (Figure 4) and of these and additional, species-specific parameters for the engraftment of individual donor strains (Figure 5).

Overall donor microbiota engraftment (Figure 4) was calculated as the donor-derived strain fraction of the total number of donor-derived and recipient-derived strains per post-FMT sample. By calculating strain fractions, our donor microbiota engraftment metric reduces confounding effects, as variable sequencing depths affect the total number of detectable strains per sample. As a consequence, the ratio of donor-derived to recipient and donor-derived strain numbers is reflective of the degree to which the recipient microbiota is replaced by the donor microbiota after FMT, whereas donor-derived strain numbers alone would not be able to differentiate microbiota replacement from microbiota expansion, i.e. an engraftment of donor strains on top of persisting recipient strains. Donor-derived post-FMT strain fractions were modeled (GLMM, binomial distribution, logit link) across the entire meta-cohort by incorporating centered and scaled microbiota and clinical parameters as fixed effects and by controlling for repeated patient sampling and study effects by including case and study as random effects. Marginal effects were calculated with ggeffects v1.1.0 (ggpredict function[82]) and model coefficients visualized in a forest plot with sjPlot v2.8.8.[83] The resulting model outputs are tabulated in Tables S5 and S6. We additionally applied gradient-boosted decision trees with xgboost v1.4.1.1 (xgboost function[84]) using five-fold cross-validation to capture non-linear associations in the data (Figure S3). Data were split into training and test sets (80/20% split), further blocking individual cases to avoid data leakage during training. We calculated SHAP (Shapley additive explanations)

values to visualize features that were important in the modeling outcome. SHAP values represent the change in the modeled output variable that varies depending on specific individual features.

We expanded our model to estimate the relevance of relative abundances and functional features (Gram stain, spore formation, oxygen tolerance, oral habitat) on the engraftment probabilities of individual strains in the meta-cohort (except Ng et al. for which patient/donor pairing information was not available). Using all species that were detected in a donor and provided only with donor and pre-FMT patient microbiota information, the model predicted whether the corresponding donor-derived strain would be detected in the post-FMT patient or not. Functional features were coded on the species level as +1 (yes, positive) and −1 (no, negative), replacing missing values with each feature's mean frequency across all species (in order to avoid an influence on the model). Since some donors were used to treat multiple patients, in addition to repeated sampling and study effects, we also controlled for donor-specific influences with random effects in our model. The adjusted engraftment probability of each genus (Table S6) is shown in the context of a phylogenetic tree (Figure 5A, ggtree v1.16.6[85,86]) that is annotated with lifestyle features for each species, and as a point range (±s.e.), with probabilities of each genus additionally conditioned on the minimum and maximum relative abundance observed within the donor population (Figure 5B).

The GLMM for the engraftment of individual strains (binomial distribution, logit link) closely fit the underlying data, both for predicted donor strain engraftment events of individual genera and aggregating total numbers of engrafted strains ($r = 0.94$, $p < .0001$). Therefore, we used the model to simulate FMT outcomes for other recipient/donor pairs from the meta-cohort. For these simulations, species-level taxonomic compositional profiles of recipient and donor samples, as determined with MetaPhlAn3, were used as input for the GLMM and the predicted total number of engrafted donor strains (based on individual predictions for each donor strain) were aggregated for each patient and time point and compared to actually detected numbers from the real recipient/donor pairs by calculating (log2) fold-changes (Figure 5D).

ADDITIONAL RESOURCES

The code for SameStr, including documentation, examples and notebooks, is available at Github (https://www.github.com/danielpodlesny/SameStr).

Cell Reports Medicine, Volume *3*

Supplemental information

Identification of clinical and ecological determinants of strain engraftment after fecal microbiota transplantation using metagenomics

Daniel Podlesny, Marija Durdevic, Sudarshan Paramsothy, Nadeem O. Kaakoush, Christoph Högenauer, Gregor Gorkiewicz, Jens Walter, and W. Florian Fricke

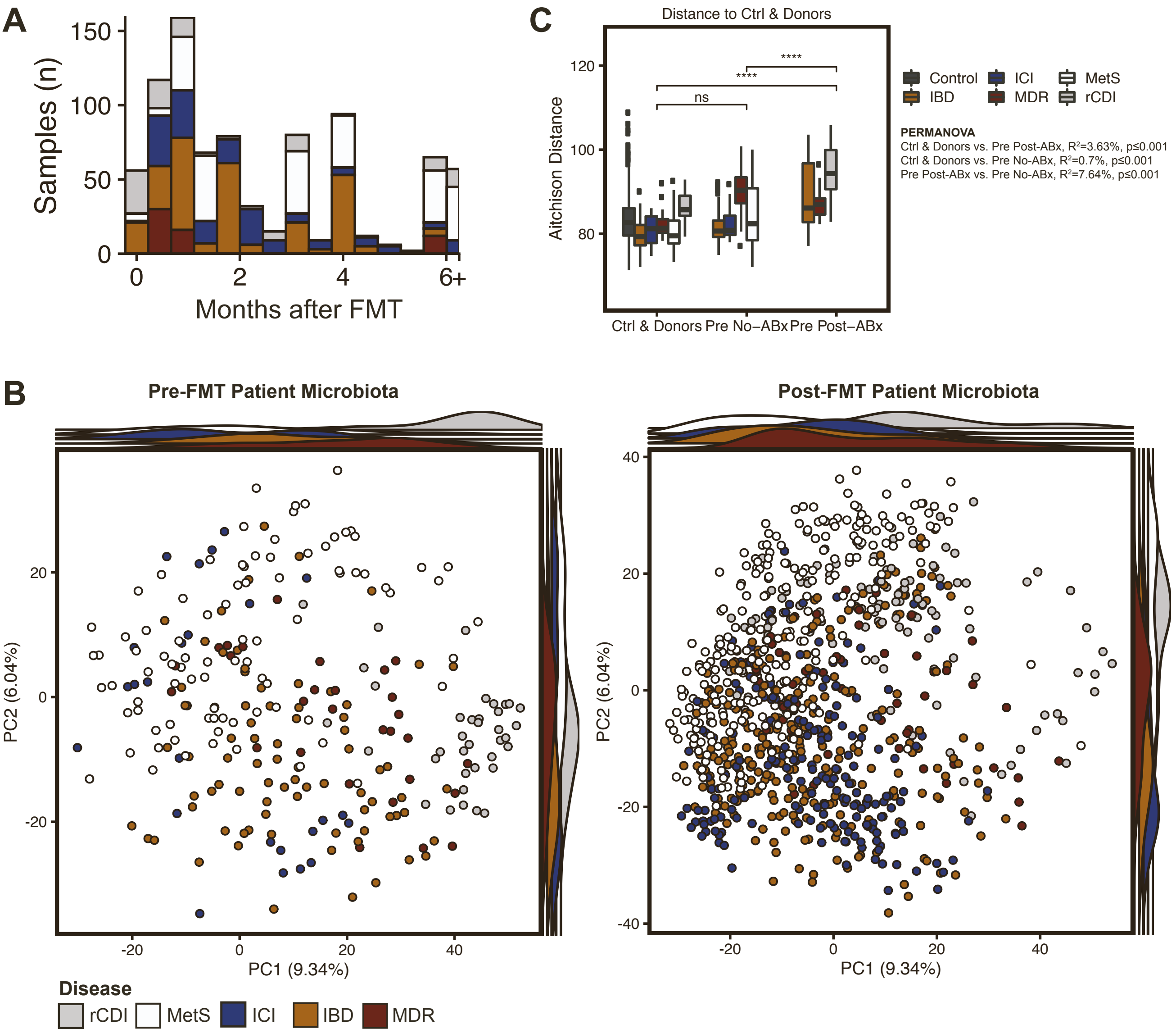

Figure S1. Overview of FMT samples between disease categories. Related to Figure 1. A. Available post-FMT samples and the time points of their collection. Histograms show the number of post-FMT samples that were collected during different time periods after FMT, colored by disease categories. B. Patient taxonomic microbiota composition across disease conditions. Taxonomic microbiota compositions of Pre-FMT and Post-FMT patients based on principal component analysis (PCA) of centered log-ratio-transformed relative species abundance, colored by disease background. Pre-FMT time points include only samples collected from patients before antibiotic treatments as part of the patient preparation for FMT, although rCDI patients received antibiotics due to their disease history. C. Taxonomic microbiota composition before and after antibiotic pretreatment. P-values based on the pairwise Wilcoxon test of Aitchison distances to Control and Donor samples (left) with FDR (BH) correction for multiple hypothesis testing, and PERMANOVA between groups (perm=999, adonis function, right). Asterisks denote significance thresholds: *p≤.01, **p≤.001, ***p≤.0001.

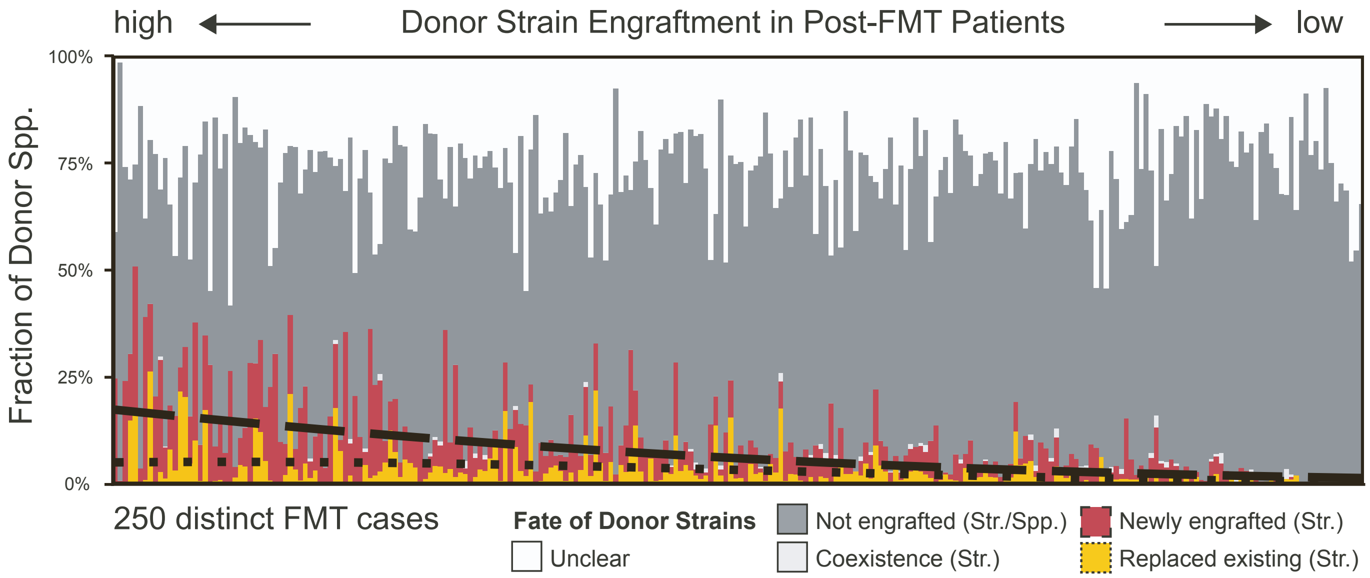

Figure S2. Fate of donor strains after FMT treatment. Related to Figure 3. Fate of donor strains after FMT treatment as the fraction of species detected in the donor microbiota, showing the same 250 FMT cases and sorting as depicted in Fig. 3A. Strains from the majority of donor species did not engraft in the patient (dark gray), and engrafted donor strains more often represented newly introduced species (red, dashed line) than replaced patient strains from a species that pre-existed in the patient before FMT (yellow, dotted line). Lines show binomial smoothing of the Fraction of Donor Spp. (glm, logit link).

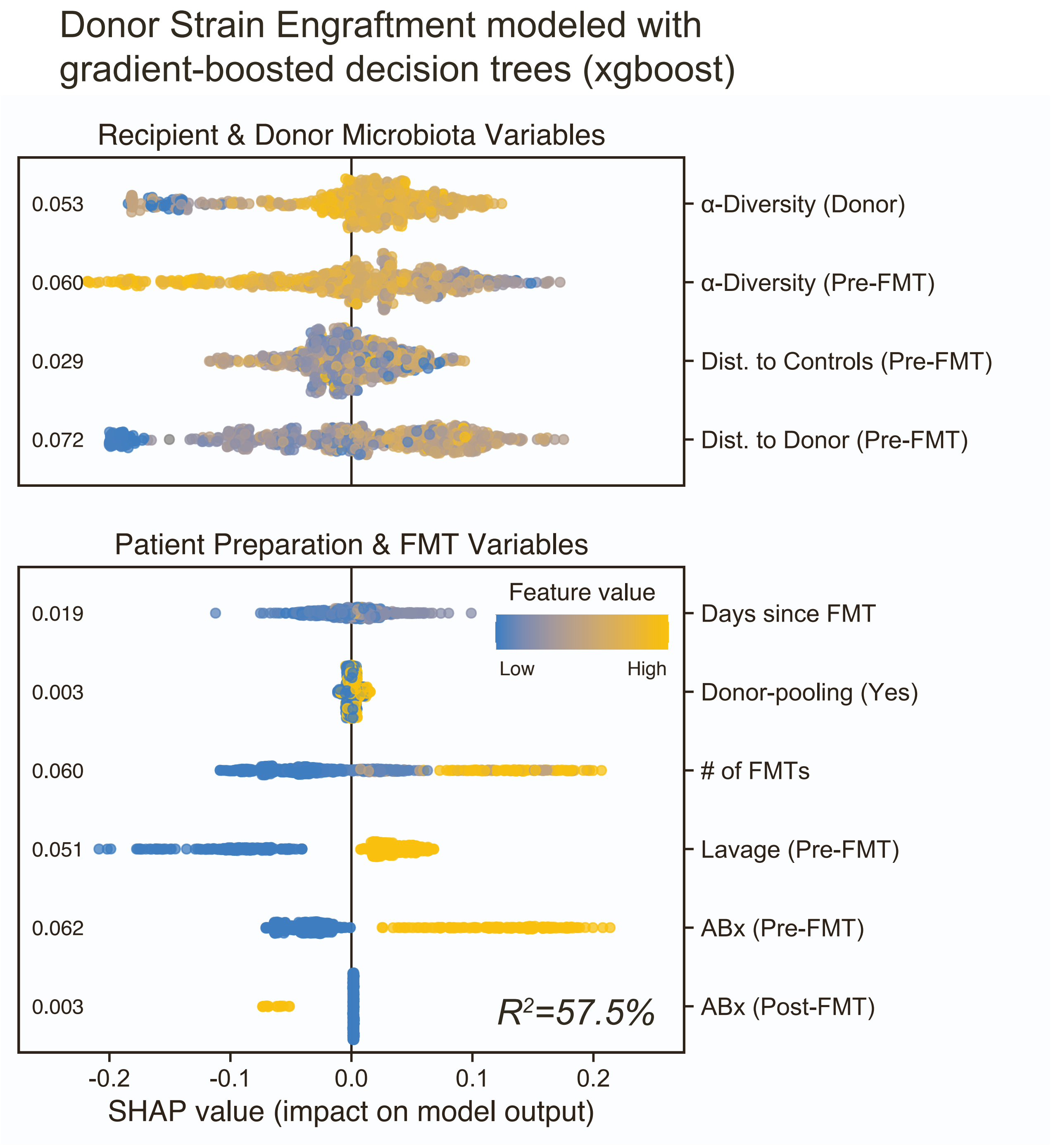

Figure S3. Gradient-boosted decision trees predict donor strain engraftment. Related to Figure 4. Gradient-boosted decision trees implemented in xgboost were applied to model donor strain engraftment as shown in Fig. 4. Data split into training and test set (80/20% split) was further blocked for individual cases to avoid information leakage during training. Model predictions based on fivefold cross-validation correlated strongly with hold-out data (r=0.76, p<.001). SHAP (Shapley additive explanations) values were calculated to visualize feature impact on model output.

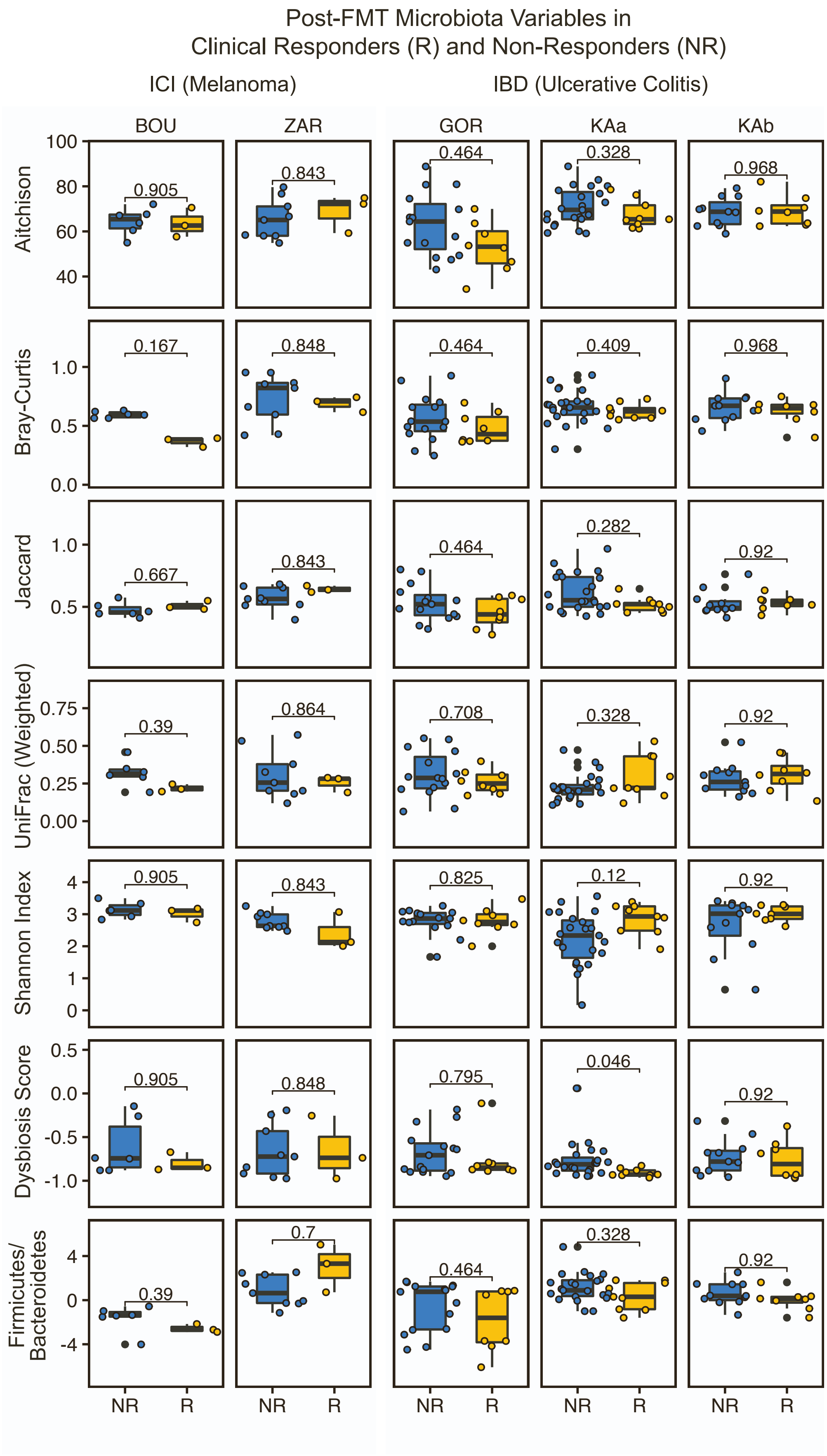

Figure S4. Microbiota comparisons between responders (R) and non-responders (NR) in ICI and IBD trials. Related to Figure 6. α-diversity (Shannon Index), Dysbiosis Score, the Firmicutes/Bacteroidetes ratio, and β-diversity (Aitchison distance, Bray-Curtis dissimilarity, Jaccard distance, weighted UniFrac) between patients and their donors for Responder/Non-Responder patient groups (see Methods) within the ICI and IBD studies. P-values based on the Wilcoxon test with FDR (BH) correction for multiple hypothesis testing within studies.

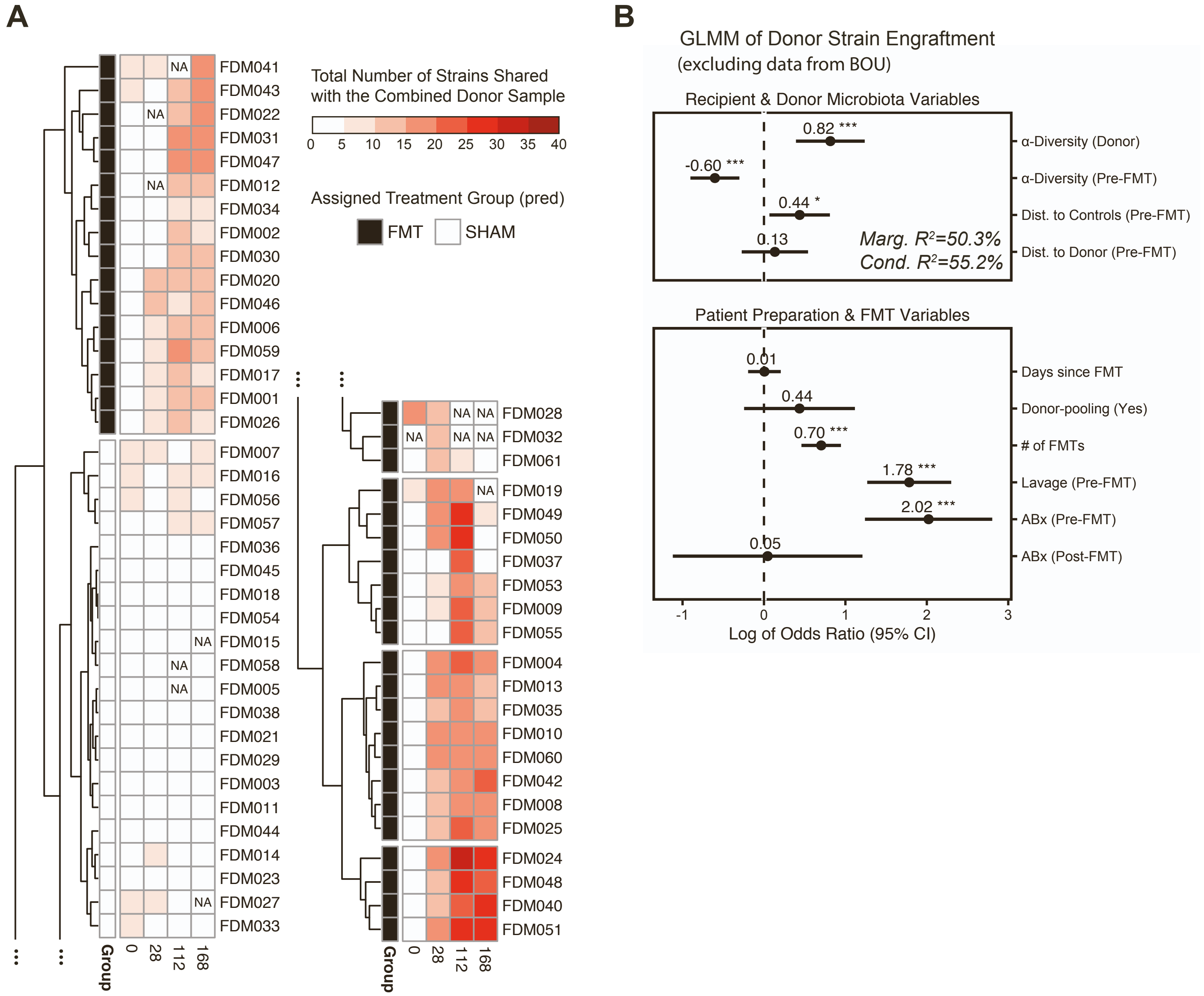

Figure S5. Data validation for the inclusion of FMT studies with missing information. Related to STAR Methods "Identification of recipient and donor-derived strains in post-FMT patients". A. Clustering of all samples from Ng et al. [1] based on shared strain profiles in order to determine the assignment of patients to the FMT or sham treatment groups. Hierarchical clustering of strain-sharing profiles between a concatenated donor sample (combined from five published donor metagenomes) and each patient from Ng et al. at one pre-FMT (0) and three post-FMT (28, 112, 168) time points highlights 21 patients whose samples share very few (mostly <5) strains with any donor sample. Since treatment assignments were not disclosed by Ng et al., these 21 patients were excluded from the analysis as they most likely belong to the sham treatment group of the study. **B.** GLMM of donor strain engraftment excluding data from Baruch et al. [2] Baruch et al. (BOU) collected pre-FMT samples before pretreatment with antibiotics. As for this dataset patient α-diversity and β-diversity to controls and donors was unavailable for the pre-FMTABx- sample, which would be most relevant for our model, the mean of those values that were observed in all other antibiotically treated pre-FMT patients was used instead to generate the model shown in Fig. 4. Here we generated a similar model without the data from Baruch et al., showing very similar results (odds ratio and significance) as in Fig. 4.

Supplementary Text S1. Influence of antibiotic used for patient pretreatment and route and number of FMT applications on donor strain engraftment. Related to Figure 3.

Different antibiotic pretreatments had distinct effects on post-FMT donor strain engraftment: MDRABx+ patients treated with antibiotics specific for Gram-negative bacteria (colistin and neomycin) acquired smaller proportions of donor-derived strains (HUT, 40.1 ± 33.6% of 209 strains) than IBDABx+ patients treated with an antibiotic cocktail against Gram-positive bacteria, parasites and fungi (vancomycin, paromomycin, and nystatin) (GOR, 94.4 ± 8.2% of 601 strains) or ICIABx+ patients treated with vancomycin and neomycin (BOU, 85.6 ± 15.7% of 232 strains) (Fig. 3C). The antibiotic treatment against Gram-positive bacteria in the IBDABx+ patients, which was associated with increased donor strain engraftment after FMT, also presented with enhanced dysbiosis before FMT (see GOR and HUT in Fig. 2).

Other forms of patient pretreatment, as well as the route of FMT application, also affected post-FMT microbiota assembly. Donor-derived strain contributions were larger in the KAa subgroup of FMT-treated IBD patients that received bowel preparation by lavage, followed by colonoscopic FMT and multiple enemas (75.6 ± 21.3% of 357 strains), compared to the KAb subgroup of the same study [3], which was treated with multiple enemas alone, without lavage and colonoscopic FMT (59.7 ± 27.5% of 298 strains) (Fig. 3C).

SUPPLEMENTAL REFERENCES

1. Ng, S.C., Xu, Z., Mak, J.W.Y., Yang, K., Liu, Q., Zuo, T., Tang, W., Lau, L., Lui, R.N., Wong, S.H., et al. (2021). Microbiota engraftment after faecal microbiota transplantation in obese subjects with type 2 diabetes: a 24-week, double-blind, randomised controlled trial. Gut.
2. Baruch, E.N., Youngster, I., Ben-Betzalel, G., Ortenberg, R., Lahat, A., Katz, L., Adler, K., Dick- Necula, D., Raskin, S., Bloch, N., et al. (2021). Fecal microbiota transplant promotes response in immunotherapy-refractory melanoma patients. Science 371, 602–609.
3. Paramsothy, S., Nielsen, S., Kamm, M.A., Deshpande, N.P., Faith, J.J., Clemente, J.C., Paramsothy, R., Walsh, A.J., van den Bogaerde, J., Samuel, D., et al. (2019). Specific Bacteria and Metabolites Associated With Response to Fecal Microbiota Transplantation in Patients With Ulcerative Colitis. Gastroenterology 156, 1440–1454.e2.
